# Climate change vulnerability and conservation strategies for tertiary relict tree species: Insights from landscape genomics of *Taxus cuspidata*

**DOI:** 10.1111/eva.13686

**Published:** 2024-09-04

**Authors:** Yanjun Luo, Wei Qin, Yu Yan, Kangquan Yin, Runguo Zang, Fang K. Du

**Affiliations:** ^1^ School of Ecology and Nature Conservation Beijing Forestry University Beijing China; ^2^ School of Grassland Science Beijing Forestry University Beijing China; ^3^ Key Laboratory of Forest Ecology and Environment, the State Forestry Administration Institute of Forest Ecology, Environment and Protection, Chinese Academy of Forestry Beijing China

**Keywords:** climate change, conservation, genetic diversity, genotype‐environment associations, habitat fragmentation, local adaptation

## Abstract

The unprecedented habitat fragmentation or loss has threatened the existence of many species. Therefore, it is essential to understand whether and how these species can pace with the environmental changes. Recent advantages in landscape genomics enabled us to identify molecular signatures of adaptation and predict how populations will respond to changing environments, providing new insights into the conservation of species. Here, we investigated the pattern of neutral and putative adaptive genetic variation and its response to changing environments in a tertiary relict tree species, *Taxus cuspidata* Sieb. et Zucc, which is distributed in northeast China and adjacent regions. We investigated the pattern of genetic diversity and differentiation using restriction site‐associated DNA sequencing (RAD‐seq) and seven nuclear microsatellites (nSSRs) datasets. We further explored the endangered mechanism, predicted its vulnerability in the future, and provided guidelines for the conservation and management of this species. RAD‐seq identified 16,087 single nucleotide polymorphisms (SNPs) in natural populations. Both the SNPs and nSSRs datasets showed high levels of genetic diversity and low genetic differentiation in *T. cuspidata*. Outlier detection by *F*_ST_ outlier analysis and genotype‐environment associations (GEAs) revealed 598 outlier SNPs as putative adaptive SNPs. Linear redundancy analysis (RDA) and nonlinear gradient forest (GF) showed that the contribution of climate to genetic variation was greater than that of geography, and precipitation played an important role in putative adaptive genetic variation. Furthermore, the genetic offset and risk of non‐adaptedness (RONA) suggested that the species at the northeast edge may be more vulnerable in the future. These results suggest that although the species has maintained high current genetic diversity in the face of recent habitat loss and fragmentation, future climate change is likely to threaten the survival of the species. Temperature (Bio03) and precipitation (Prec05) variables can be potentially used as predictors of response of *T. cuspidata* under future climate. Together, this study provides a theoretical framework for conservation and management strategies for wildlife species in the context of future climate change.

## 1 INTRODUCTION

Habitat loss or fragmentation due to climate change or human activity is recognized as a major threat to global biodiversity (Pykälä, 2019; Rands et al., 2010). Among the various forms of biodiversity, genetic diversity is particularly important in conservation biology, as it provides the raw material for evolutionary change and thus has the potential to adapt to changing environments (Laikre et al., 2010; Nielsen et al., 2022; O'Brien et al., 2022). Normally, genetic diversity might decrease because of increased random genetic drift, inbreeding, and reductions in gene flow due to habitat loss or fragmentation of species (Aguilar et al., 2008; Chung et al., 2023; Lowe et al., 2005; Young et al., 1996). However, habitat loss or fragmentation can also lead to highly specific genetic consequences (e.g., increased genetic divergence, genetic bottleneck and reduced effective population size) for plants because of their different life histories, particularly for tree species with long generation times (e.g., González et al., 2020; Petit & Hampe, 2006; Piotti, 2009).

Forest tree species are largely undomesticated and exhibit local adaptation across heterogeneous environments (Bonan, 2008; Litkowiec et al., 2018). Neutral molecular markers, such as microsatellite genotyping, chloroplast DNA (cpDNA) and mitochondrial DNA (mtDNA) fragments, have been used to reveal the genetic dynamics of forest tree species especially endangered tree species. In general, low genetic diversity and strong genetic differentiation were revealed in endangered or critically endangered tree species with small population size because of the enhanced genetic drift effect and inbreeding (e.g., Frankham, 2005; Yang et al., 2020), such as *T. yunnanensis* (Miao et al., 2016), *Abies ziyuanensis* (Tang et al., 2008), *Cathaya argyrophylla* (Wang & Ge, 2006). However, high levels of genetic diversity have also been detected in some endangered tree species with current small or scattered population size (e.g., de Sousa et al., 2020; Gitzendanner et al., 2012; Yousefzadeh et al., 2018).

To date, neutral molecular markers are the most common tools for conservation genetic studies, however, genetic variation at neutral loci cannot provide direct information on adaptive selective processes involving the interactions between individuals and their environment (Hoffmann & Willi, 2008; Holderegger et al., 2006). Recently, with the advent of high‐throughput sequencing technologies, adaptive genetic markers have become available even for tree species with large and complex genomes (Feng & Du, 2022; Mackay et al., 2012; Neale & Kremer, 2011). In addition, emerging landscape genomics aims to detect adaptive variation along the landscape by integrating geographic and environmental information to provide unprecedented insights into the evolutionary mechanisms of local adaptation (Borrell et al., 2020; Du et al., 2020; Sork et al., 2013; Wang et al., 2021). *F*_ST_ outlier analysis and genotype‐environment associations (GEAs) in landscape genomics have been suggested as promising tools for detecting adaptive genetic variation in conservation practices (Ahrens et al., 2018; Chung et al., 2023; Schoville et al., 2012). In recent years researchers have increasingly employed these methods to study how spatial heterogeneity promotes population genetic dynamics, and how genomic variation promotes adaptive evolution in forest tree species, particularly conifers (Jia et al., 2020; Mayol et al., 2020; Zhao et al., 2020).

*Taxus cuspidata* Sieb. et Zucc., a tertiary relict tree species, is a keystone‐dominant coniferous long‐lived wind‐pollinated tree species with a discontinuous distribution in Japan, Korea, Northeast (NE) China and Far Eastern Russia (Kunashir Island, Sakhalin and Primorye) (Chung et al., 1999; Kitamura & Murata, 1987). In China, the species is mainly located at altitudes ranging from 600 m to 1200 m in the Changbai Mountains and neighboring areas. The number of natural populations of *T. cuspidata* has drastically decreased in the last century, partially because of human disturbance. Therefore, *T. cuspidata* is considered as “Plant Species with Extremely Small Populations (PSESP)” in China (Wade et al., 2016), however, it was a Least Concern (LC) species in the International Union for Conservation of Nature (IUCN) (Katsuki & Luscombe, 2013). Previous studies based on paternal inherited mtDNA and cpDNA fragments have shown high degrees of genetic diversity in *T. cuspidata* (Cheng et al., 2015; Kozyrenko et al., 2017; Su et al., 2018). However, the contributions of geography and climate to genetic variation in *T. cuspidata* remain unclear.

Here, we sampled *T. cuspidata* natural populations throughout the species' distribution range and combined their neutral and non‐neutral genetic variation using nSSRs and SNPs. We aimed to (1) estimate the genetic diversity and genetic differentiation within and among populations, (2) understand the contribution of geographic and climatic factors to genetic variation and (3) predict future adaptability of the species using landscape genomic tools under a scenario of future climate change. Our study lays the groundwork for understanding the molecular mechanisms of local adaptation, and provides a theoretical foundation for the conservation and management of important conifer tree species in East Asia.

## 2 MATERIALS AND METHODS

### 2.1 Field sampling

In our study, we collected leaf material in natural populations of *T. cuspidata* in the Changbai Mountains and adjacent areas. The sampling points were strategically distributed across the area, spanning from the southwest to the northeast of Changbai Mountains and adjacent areas. A total of 200 individuals of *T. cuspidata* were sampled from 19 natural populations and each population was separated by at least 30 km. The detail sampling information of each population was listed in Table S1. Leaf samples were labeled and stored in plastic bags with silica gel for DNA extraction. Voucher specimens of each population were deposited at Beijing Forestry University.

### 2.2 DNA isolation and microsatellite genotyping

Total genomic DNA was extracted from leaf tissue using Plant Genomic DNA Kit (Tiangen, Beijing, China) following the manufacturer's protocol. The DNA quality was initially checked by 1% agarose electrophoresis gel and then the concentration was measured by an ultramicro‐spectrophotometer (Thermo Fisher, USA). Seven polymorphic nuclear microsatellite (nSSR) loci were used for genotyping all 200 individuals after initial screening of 24 nSSR primers that had been already applicable to *T. cuspidata* (Cheng et al., 2015; Dubreuil et al., 2008; Kondo, 2016) (see Table S2 for primer details). The PCR conditions followed Du et al., 2017 and the PCR products were analyzed using an ABI PRISM 3730 Genetic Analyzer (Applied Biosystems, USA). Subsequently, the alleles were scored using GENEMARKER v. 2.2 (Softgenetics, USA) and the genotype was checked twice.

### 2.3 Double‐digest RADseq‐derived SNP dataset

A subset of samples (130 out of 200 individuals in 19 populations) was used for SNP dataset collection by a double‐digestion restriction fragment‐based procedure (ddRAD‐seq) (Peterson et al., 2012) (Table S3). The subset of sampling by RAD‐seq covered almost the entire distribution range to capture the vast majority of diversity. For each sample, the genomic DNA was processed for library construction and sequencing. Briefly, DNA was double‐digested with restriction enzyme *Taq*I and *Mse*I, followed by the ligation of Illumina adaptors. Ligation products were size‐selected about 500 bp and amplified by kapa HotStart ReadyMix (cat. no. KK2601; KAPA Biosystems) with 13 cycles. Paired‐end sequencing (2 × 150 bp) was performed using an Illumina HiSeq2000 platform at Majorbio Pharm Technology Co., Ltd., Shanghai, China.

The data quality control was assessed by FastQC v0.11.7 (Andrews, 2010). We removed adapter sequences and low‐quality bases (Phred quality <20) from raw data using Trimmomatic 0.36 (Bolger et al., 2014). Then the reads were all trimmed to 120 bp and designed for SNP genotyping using Stacks v1.48 (Catchen et al., 2011, 2013). In detail, *ustacks* was used to assemble our sequences into de novo, *cstacks* was used to build a catalog of loci, *sstcaks* was used to match loci against the catalog and the *Populations* was used to output SNPs. A strict criteria was used to filter SNPs using *VCFtools* (Danecek et al., 2011): first, we only kept individuals that represented at least 60% of the SNPs; second, a filtering parameter of 0.8 was used to avoid the influence of missing data, i.e., more than 80% of individuals in a population were required to process a locus; third, a minor allele frequency (MAF) < 0.1 was used to filter the data and to reduce the likelihood of false‐positive results due to spurious correlations.

### 2.4 Genetic diversity and structure

We estimated genetic diversity including mean effective number of alleles (*N*_E_), mean observed heterozygosity (*H*_O_), mean expected heterozygosity (*H*_E_) and mean number of different alleles (*N*_A_) for each population by GenAlEx 6.5 for nSSR dataset (Peakall & Smouse, 2006). We defined two types for ddRAD‐seq dataset: neutral genetic variation based on neutral SNPs and non‐neutral (putatively adaptive) genetic variation based on outlier SNPs (i.e., outliers were detected by *F*_ST_ and GEA analysis; see section outlier detection below). We estimated the mean frequency of the most frequent allele at each locus (*P*), mean nucleotide diversity (*π*), *H*_O_ and *H*_E_ for all, neutral, *F*_ST_‐outlier and GEA‐outlier SNPs by *Populations* module in Stacks (Catchen et al., 2013).

We conducted a principal component analysis (PCA) to produce a lower dimensional subspace that captured most of the variation in each of three data types. For nSSRs, “adegenet” (Jombart & Ahmed, 2011) was used for the PCA in R 3.6.1 (R Core Team, 2019). PLINK v.1.07 was used for the PCA in all, neutral and outlier SNPs (Purcell et al., 2007; Zheng et al., 2012).

Population structure was also accessed using Bayesian clustering. For nSSRs, we identified population genetic structure through the Bayesian Markov chain Monte Carlo (MCMC) clustering method implemented in STRUCTURE v 2.3.4 (Pritchard et al., 2000). Twenty independent runs were performed for each value of *K* (1–10) using 200,000 generations for the MCMC cycles and 100,000 generations for the burn‐in by STRUCTURE HARVESTER (Earl & vonHoldt, 2012). The most likely number of clusters (K) was determined using ΔK and LnP(K) statistics, according to Evanno et al. (2005). CLUMPP (Jakobsson & Rosenberg, 2007) and DISTRUCT v1.1 (Rosenberg, 2004) were used to aligned independent runs and visualize the bar plots of the individual's probabilities of population membership.

ADMIXTURE v1.3.0 (Alexander et al., 2009; Alexander & Lange, 2011), which can deal with large data with fast speed, was applied to assess population structure of genetic variation for all, neutral and outlier SNPs. We ran ADMIXTURE with default 5‐fold cross‐validation (−cv = 5) to select the optimal K from one to ten, the best K exhibits low cross‐validation error (CV error) opposed to others (Alexander & Lange, 2011).

We conducted a hierarchical analysis of molecular variance (AMOVA) to quantify the degree of genetic divergence among and within populations for our nSSRs, all SNPs, neutral SNPs, *F*_ST_‐outlier SNPs and GEA‐outlier SNPs by Arlequin 3.5 (Excoffier & Lischer, 2010). The significance of genetic differentiation differences was evaluated using 10,000 permutations.

### 2.5 Climatic variables

Bioclimatic variables of current climate (representative of 1960–1990) and future climate (2070: average for 2061–2080) were downloaded from the WorldClim Version2 (http://worldclim.org/version2, Fick & Hijmans, 2017) at spatial resolutions of 30 s (~1 km^2^). A total of 31 climate variables were extracted by longitudes and latitudes of population sites based on raster layers by ArcMap10.2. To avoid biased estimates of model coefficients and spurious significance levels resulting from multicollinearity, we excluded highly correlated climate variables with the threshold values of 0.7 using a variance inflation factor (VIF) test in “usdm” R package (Marquaridt, 1970; Naimi et al., 2014; R Core Team, 2019). Six climate variables were finally retained: isothermality (bio03), maximum temperature of warmest month (bio05), mean temperature of driest quarter (bio09), precipitation seasonality (bio15), precipitation in May (prec05) and precipitation in October (prec10). We also carried out Shapiro–Wilk and Levene test (package car, Fox & Weisberg, 2019) to explore each variable's data distribution and homogeneity. We found that the data for each variable was not normally distributed nor homogeneous; therefore, we performed the Kruskal‐Wallis rank sum test to explore the significance level for each variable.

### 2.6 Outlier detection

Three algorithms were used to detect potentially adaptive loci, including one *F*_ST_ outlier analysis approach and two genotype‐environment associations (GEAs) outlier detection approaches.

For the *F*_ST_ outlier analysis, we use Bayesian approach in BayeScan 2.0 to directly estimate the posterior probability of a given locus under selection (Foll & Gaggiotti, 2008). We used the following parameter values: sample size of 5000, 20 pilot runs with 5000 run length, 50,000 burn‐in iterations and thinning interval of 10. Prior odds for neutral model were set to 10 and SNPs with *q* < 0.01 (−log_10_ *q* > 2) were considered as *F*_ST_‐outliers (Puebla et al., 2014).

For genotype‐environment associations (GEAs) outlier detection approaches, we firstly applied latent factor mixed models (LFMM) by package LEA in R (R Core Team, 2019). We use a hierarchical Bayesian mixed modelling approach to identify allele–environment correlations, while modelling residual population structure with ‘latent factors’ (Frichot et al., 2013; Frichot & François, 2015). In LFMM, environmental variables were tested separately and introduced into each model as fixed effects, and the number of latent factors (*K* = 3) was selected to account for genetic structure by sparse non‐negative matrix factorization (SNMF) (Figure S7). The parameters were set as follows: 100,000 iterations with a burn‐in of 50,000 iterations and ten replicate runs. Significant outliers were determined as SNPs with *p*‐values of *p* < 10^−3^ or –log_10_ (*p*‐value) > 3. We secondly used Bayesian generalized linear mixed models (BayEnv) to detect the correlation between SNPs and environmental variables with a neutral dataset generated by BayeScan as a null model (Günther & Coop, 2013). We initially computed a null covariance matrix of relatedness between populations, over 100,000 iterations and five independent runs. We then tested all SNPs (including those initially identified by BayeScan) under an alternative model where allele frequencies are determined by a combination of the covariance matrix and an environmental variable. We performed our analysis independently across six environmental variables, and using Bayes factor (BF) to evaluate the posterior probability that each SNP is under selection across independent environmental variable. We also performed nonparametric Spearman's rank correlation as alternative tests to the BF and detect the correlation between ranks of SNP allele frequencies and environmental variables. Therefore, we considered the SNPs in the top 1% of BF values (BF > 3) and top 10% of the absolute value of Spearman rank correlation coefficients (*ρ*) as significant outliers.

### 2.7 Multivariate relationship between genetic variation and environmental gradients

To estimate how the genomic variation is influenced by climate or geographic variables, we firstly performed redundancy analyses (RDAs) and partial redundancy analyses (*p*RDAs) to detect linear relationships between genetic variations and multivariate climatic gradients, using “vegan” R package (Oksanen et al., 2019). We constructed two *p*RDAs models to differentiate the independent effects of climate and geography by reciprocally constraining one of the two factors. Briefly, climatic effects were conditioned on the effects of geography (*p*RDAenv to extract pure effects of the climate) and vice versa (*p*RDAgeo for pure effects of geography). All SNPs, neutral and non‐neutral genetic variation with the six climate variables and geographic variables (longitude and latitude) were considered as predictor variables. Statistical significance was evaluated from 999 permutations.

We then employed gradient forest (GF) analyses to explore the nonlinear relationships between environmental variables and their contribution to genetic variation using R package GradientForest (Ellis et al., 2012). Gradient forest is a machine learning, regression tree approach that allows for exploration of nonlinear associations of spatial, environmental and allelic variables (Ellis et al., 2012; Fitzpatrick & Keller, 2015). We conducted a GF analysis on six climate variables to assess the relative importance of each predictor variable using weighted R^2^ value, split importance; Ellis et al. (2012). Split importance, a measure of the amount of variation explained, is high in positions along the gradient where allelic change is large. We ran a gradient forest with 500 regression trees per SNP, maxLevel = log_2_(0.368n)/2, and variable correlation threshold of 0.5 to calculate conditional variable importance as recommended (Ellis et al., 2012; Fitzpatrick & Keller, 2015). The default values of the other parameters were carried out for each GF model.

### 2.8 Adaptation potential of *Taxus cuspidata*

We employed two different methods to estimate the adaptive potential of *T. cuspidata*. We selected the Global Climate Model BCC‐CSM1‐1 (IPCC, 2014) under two contrasting representative concentration pathways (RCPs), including a low‐emission scenario (RCP 2.6) and a high‐emission scenario (RCP 8.5) in 2070 years for future climate scenarios. We first used GF to estimate the genetic offset based on all SNPs, *F*_ST_‐outlier SNPs and GEA‐outlier SNPs under future climatic conditions. Genetic offset, a measure of the magnitude of genetic change required between present and future climate (Fitzpatrick & Keller, 2015) was used to predict genetic variation across grid cells of NE China by “GradientForest” package (Ellis et al., 2012) in R (R Core Team, 2019). For each grid cell, the Euclidian distances between the current and future genetic compositions were calculated and served as the metric for genetic offset (Ellis et al., 2012).

We also performed a risk of non‐adaptedness (RONA) to predict the adaptive potential of *T. cuspidata* to future local climate using default settings in PYRONA v0.3.6 (Pina‐Martins et al., 2019; Rellstab et al., 2016). PYRONA ranked the environment factors by the number of associations and provided an average RONA value weighted by the regression R^2^ value for each predictor factors. The “RONA value” here indicates the mean difference between current and future expected allele frequencies, which was used to estimate the expected allele frequencies in future based on the present allele frequencies and environmental variables. The inferred linear model was used to predict expected allele frequencies in future environmental gradients in 2070 according to the Global Climate Model BCC‐CSM1‐1 (IPCC, 2014) under RCP 2.6 and RCP 8.5.

## 3 RESULTS

### 3.1 RAD‐based SNP data

We attained a total of 373,894,701 loci with the mean depth of coverage for filtered SNPs at 4.89 (range: 3.58–7.47) and mean proportion of loci with missing data per sample is 0.46 (range: 0.07–0.98) (Table S3). A total of 37 individuals with high proportion missing data were discarded, and the retaining 93 individuals is at least four individuals per population (Table 1). Stacks initially recovered 144,575 SNPs and 16,087 high‐quality SNPs were retained after stringent quality control.

**TABLE 1:** Genetic diversity of *Taxus cuspidata* based on neutral and non‐neutral genetic variation.

		Neutral genetic variation								Non‐neutral genetic variation							
		nSSRs				Neutral SNPs				*F*_ST_‐outlier SNPs				GEA‐outlier SNPs			
Population	*N*	*N*_A_	*N*_E_	*H*_o_	*H*_E_	P	*H*_o_	*H*_E_	π	P	*H*_o_	*H*_E_	π	P	*H*_o_	*H*_E_	π
MDJHCH	15 (8)	4.14	2.48	0.39	0.48	0.79	0.27	0.28	0.30	0.86	0.18	0.19	0.21	0.77	0.27	0.32	0.34
MDJS	15 (8)	4.14	2.34	0.36	0.44	0.78	0.25	0.30	0.32	0.77	0.16	0.30	0.33	0.77	0.25	0.31	0.33
HCM	15 (8)	3.43	1.90	0.29	0.40	0.80	0.26	0.27	0.30	0.81	0.31	0.25	0.27	0.75	0.29	0.33	0.35
MDJHP	15 (7)	3.57	2.17	0.38	0.43	0.80	0.24	0.26	0.29	0.87	0.15	0.17	0.18	0.78	0.25	0.29	0.31
YBD	15 (8)	3.57	2.13	0.42	0.46	0.79	0.28	0.29	0.31	0.80	0.29	0.29	0.31	0.76	0.31	0.32	0.34
YBHG	15 (8)	3.00	1.90	0.29	0.34	0.78	0.31	0.30	0.32	0.84	0.23	0.22	0.24	0.76	0.34	0.32	0.35
YBJ	5 (4)	2.29	1.71	0.46	0.34	0.81	0.31	0.26	0.30	0.88	0.19	0.17	0.20	0.79	0.33	0.28	0.33
YJX	15 (8)	2.57	1.58	0.26	0.29	0.78	0.30	0.30	0.32	0.83	0.23	0.23	0.25	0.76	0.31	0.32	0.35
YBHS	4 (4)	2.14	1.73	0.39	0.34	0.79	0.28	0.27	0.33	0.84	0.20	0.21	0.26	0.77	0.30	0.29	0.35
YBHSP	6 (6)	3.14	2.21	0.36	0.39	0.80	0.28	0.27	0.31	0.75	0.26	0.32	0.35	0.76	0.32	0.31	0.36
LJD	7 (7)	2.71	2.23	0.31	0.47	0.79	0.26	0.29	0.31	0.77	0.29	0.32	0.34	0.77	0.27	0.31	0.34
LJB	5 (4)	2.43	1.71	0.43	0.34	0.79	0.32	0.28	0.32	0.79	0.30	0.29	0.33	0.77	0.37	0.31	0.36
BSSCZ	5 (5)	2.29	1.94	0.26	0.28	0.81	0.27	0.26	0.29	0.85	0.21	0.21	0.24	0.77	0.33	0.31	0.35
THL	10 (8)	2.57	1.72	0.30	0.34	0.78	0.30	0.30	0.33	0.71	0.34	0.38	0.40	0.75	0.30	0.34	0.36
Mean		2.99	1.98	0.35	0.38	0.79	0.28	0.28	0.31	0.81	0.24	0.25	0.28	0.77	0.30	0.31	0.34

Abbreviations: *N*, number of individuals used for nSSRs and RAD‐seq in parentheses; *N*_A_, No. of different alleles and *N*_E_, effective number of alleles; *H*_o_ and *H*_E_, observed and expected heterozygosity; P, mean frequency of the most frequent allele at each locus; π, mean nucleotide diversity.

### 3.2 Genetic diversity and differentiation based on nSSRs and SNP datasets

For nSSRs, observed and expected heterozygosity estimated per population ranged from 0.26 to 0.46 and 0.28 to 0.48, respectively (Table 1, Table S5). The expected heterozygosity (*H*_E_) and nucleotide diversity (π) ranged from 0.26 to 0.30 and 0.29 to 0.33 in all (Table S4) and neutral SNPs (Table 1). For *F*_ST_‐outlier SNPs, *H*_E_ and π ranged from 0.17 to 0.38 and 0.18 to 0.40, and for GEA‐outlier SNPs, *H*_E_ and π ranged from 0.28 to 0.34 and 0.31 to 0.36 (Table 1).

**FIGURE 1:**
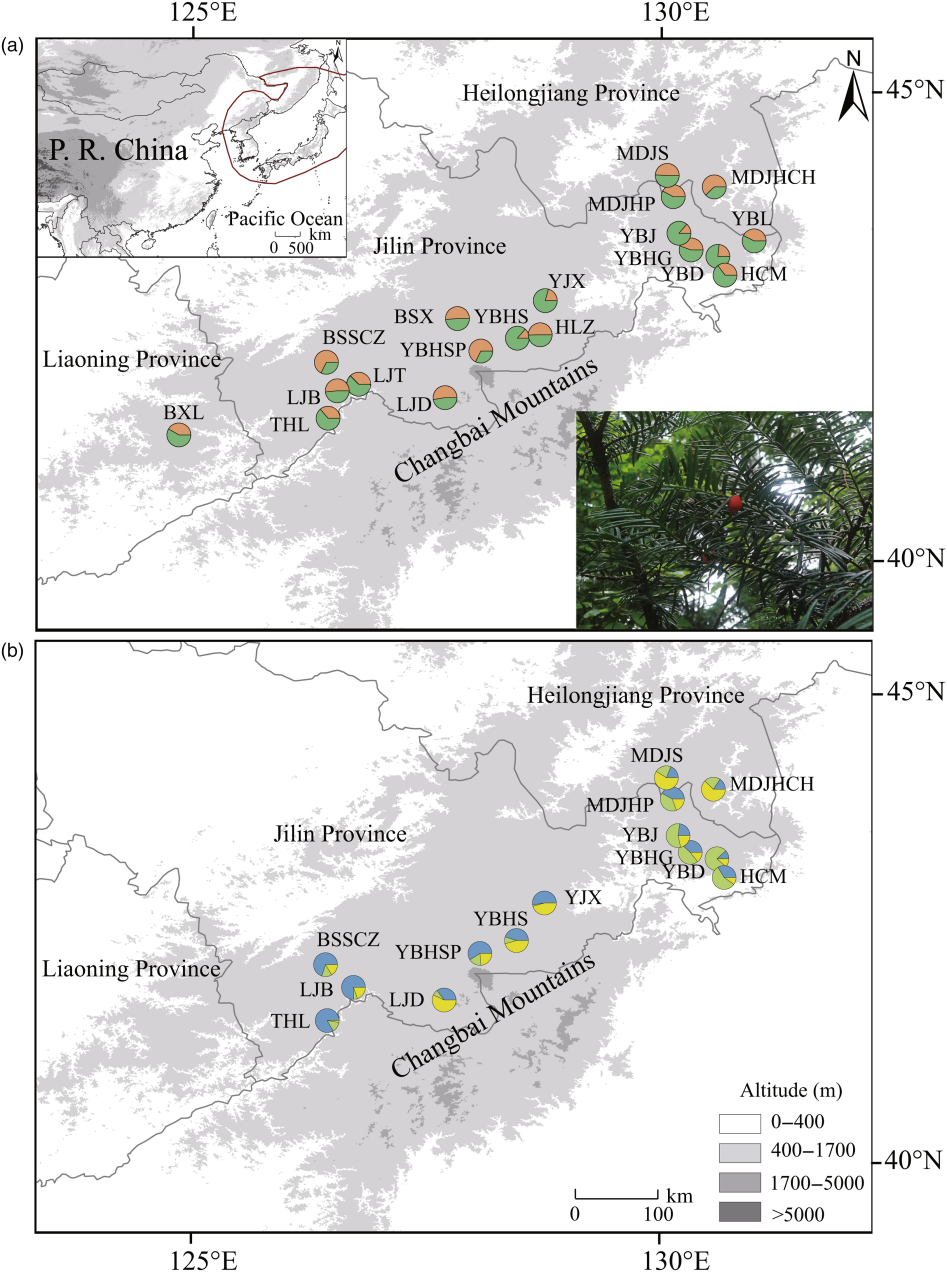
Geographic distribution and sampling sites of *Taxus cuspidata*. The pie chart shows the genetic clustering of each population based on (a) neutral genetic variation by microsatellites (SSRs) and (b) non‐neutral genetic variation by outlier SNPs. The red line indicates the distribution range of *T. cuspidata*. Orange, green, blue and yellow represent genetic cluster assignments; the details of the cluster assignments were showed in Table S1.

PCA (Figures S1, S2) based on both nSSRs and RAD‐seq datasets were consistent with Bayesian clustering analysis, which failed to detect clear population genetic structure in all populations. The most likely number of clusters (K) was two for the nSSRs (Figure 1a, Figures S3, S4), one for all and neutral SNPs (Figure 5b, S5a), and three for outlier SNPs (Figure 1b, Figures S5c, S6). These results showed a mixed genetic makeup of clusters in all populations.

The results of AMOVA indicated that a large proportion of genetic variation occurred within populations, with low levels of genetic differentiation (*F*_ST_ = 0.07) in nSSRs, all SNPs and neutral SNPs; For outlier SNPs, high *F*_ST_ values (*F*_ST_ = 0.32) in *F*_ST_‐outlier SNPs and moderate *F*_ST_ values (*F*_ST_ = 0.1) in GEA‐outlier SNPs (Table 2).

**TABLE 2:** Hierarchical analyses of molecular variance (AMOVA) of *Taxus cuspidata* populations based on all SNPs, neutral and non‐neutral genetic variation.

Source of variation	df	Percentage of variation (%)	Fixation indices
All SNPs			
Among groups	13	6.6	
Within populations	172	93.4	*F*_ST_ = 0.07
Neutral genetic variation			
nSSRs			
Among groups	18	7.5	
Within populations	381	92.5	*F*_ST_ = 0.07
Neutral SNPs			
Among groups	13	7.2	
Within populations	172	92.8	*F*_ST_ = 0.07
Non‐neutral genetic variation			
*F*_ST_‐outlier SNPs			
Among groups	13	38.5	
Within populations	172	79.1	*F*_ST_ = 0.32
GEA‐outlier SNPs			
Among groups	13	10.3	
Within populations	172	89.7	*F*_ST_ = 0.10

*Note*: Significance tests (1000 permutations) showed all fixation indices were significant (*p* < 0.05).

Abbreviation: df, degree of freedom.

### 3.3 Outlier detection and environmental association

**FIGURE 2:**
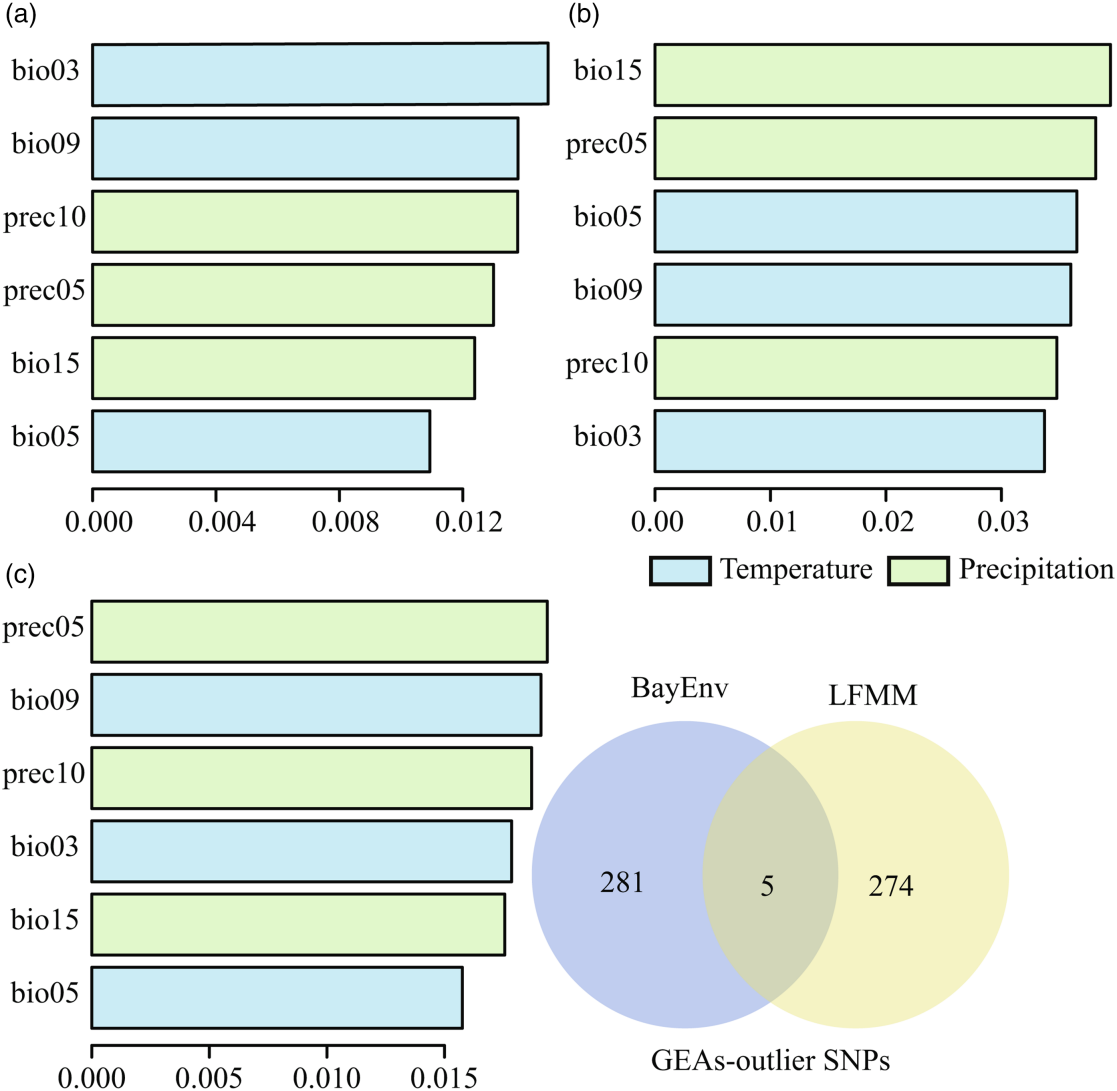
*R* ^2^‐weighted importance of environmental variables explaining genetic gradients for all (a), *F*_ST_‐outlier (b), and GEA‐outlier (c) SNPs in *Taxus cuspidata* using GF analysis. A venn diagram showing the overlap of outliers between all SNPs associated with at least one climatic variable, as identified by LFMM and BayEnv.

We identified 63 SNPs as putative outliers and did not detect any significantly low outlier *F*_ST_ values that would be indicative of balancing or purifying selection using BayeScan (Figure S8). According to the significance test, each climate variable was significantly different among the populations (Kruskal–Wallis chi‐squared = 92, *p* < 0.001). We identified 279 and 286 putative adaptive SNPs that were significantly associated with at least one climatic variable using LFMM (Figure S9) and BayEnv (Figure S10), respectively, with five common SNPs (Figure 2c). More putative adaptive SNPs were significantly associated with precipitation variables than temperature variables (Table S6). A total of 598 outlier SNPs were identified as non‐neutral genetic variations and 15,489 neutral SNPs as neutral genetic variations.

### 3.4 Environmental associations with genetic variation

RDA revealed that a large proportion of genetic variation among populations was associated with the six climate variables. The RDA results were similar to that of *p*RDA (Tables S7 and S8; Figures S11a, S12); here, we explained the results of *p*RDA (Table 3, Figures S11b, S13). For nSSRs, genetic variation was found to be mainly associated with climate variables: 5% of the nSSRs variance was explained by climate, while geography only accounted for 1% (Table 3). For all SNPs and neutral SNPs, 10% of the explained variance was explained by climate and 3% by geography (Table 3, Table S8). For outlier SNPs, 21% of the *F*_ST_‐outlier SNPs variance was explained by climate and 4% by geography; 13% of the GEA‐outlier SNPs variance was explained by climate and 3% by geography (Table 3).

**TABLE 3:** Summary and partitioning of the variance associated with climate and geographical variables based on *p*RDA in neutral and non‐neutral genetic variation.

	Neutral genetic variation						Non‐neutral genetic variation					
	nSSRs			Neutral SNPs			*F*_ST_‐outlier SNPs			GEA‐outlier SNPs		
	PVE	Eigenvalue	*p*	PVE	Eigenvalue	*p*	PVE	Eigenvalue	*p*	PVE	Eigenvalue	*p*
Geography	1.41	1.10	0.373	3.45	1.69	0.001	4.44	2.6	0.002	2.89	1.53	0.001
Climate	4.97	1.69	0.004	9.96	1.62	0.001	20.96	4.10	0.001	13.41	2.37	0.001
bio03	1.22	2.49	0.014	1.78	1.74	0.001	1.75	2.06	0.047	3.17	3.37	0.001
bio05	0.66	1.35	0.222	1.35	1.32	0.004	4.4	5.16	0.002	1.71	1.81	0.004
bio09	0.96	1.96	0.055	1.63	1.59	0.001	2.79	3.28	0.003	1.99	2.12	0.001
bio15	0.87	1.78	0.082	1.60	1.56	0.001	2.28	2.68	0.012	2.07	2.20	0.001
prec05	0.61	1.21	0.268	1.89	1.85	0.001	7.63	8.95	0.001	2.68	2.85	0.001
prec10	0.65	1.33	0.244	1.72	1.68	0.001	2.11	2.46	0.025	1.79	1.90	0.001

Abbreviation: PVE, percentage of explained variance.

For all SNPs, the GF indicated that isothermality (bio03) was the most important climate response factor (Figure 2a). However, for outlier SNPs, precipitation in May (prec05) and precipitation seasonality (bio15) were the most important climate response factors, respectively (Figure 2b,c).

### 3.5 Prediction of population vulnerability under future climate change

**FIGURE 3:**
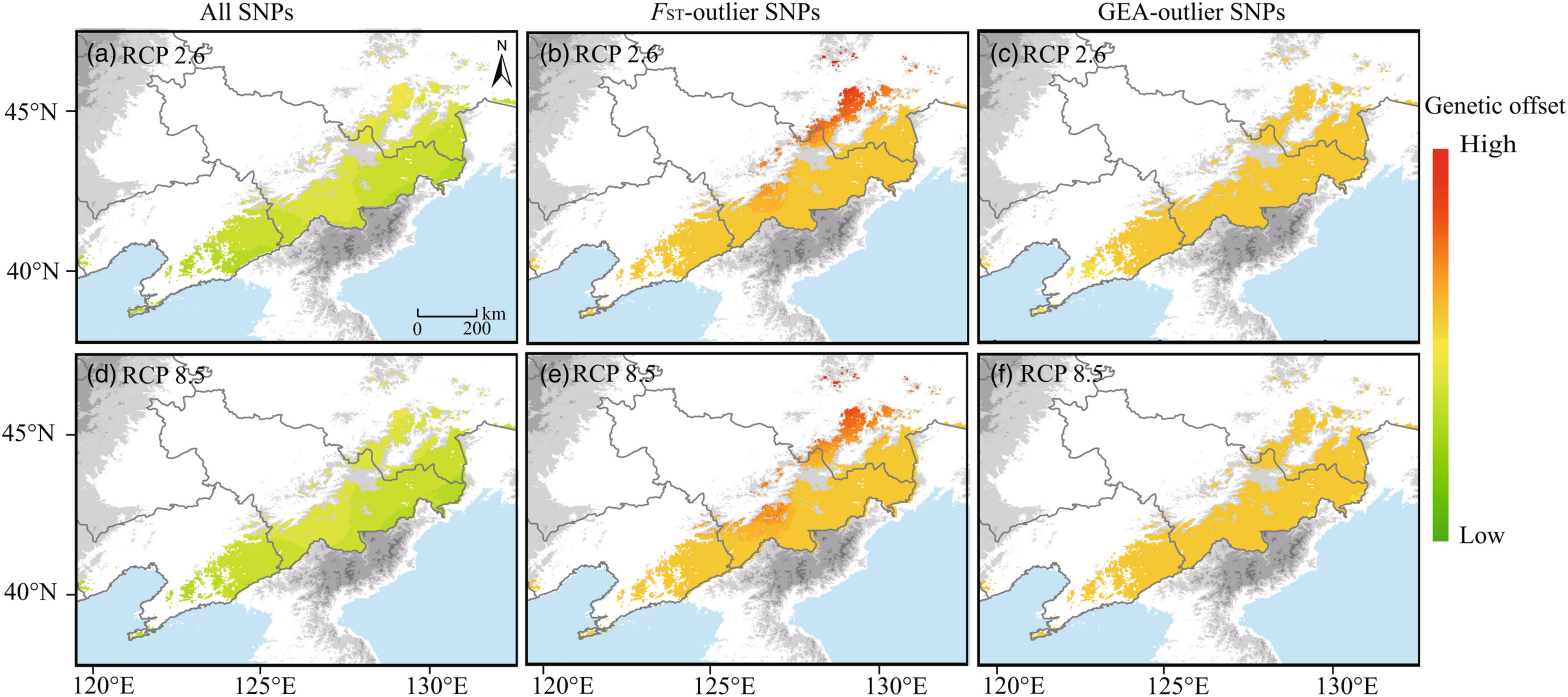
Genetic offset to future climate change predicted for all (a, d), *F*_ST_‐outlier (b, e) and GEA‐outlier SNPs (c, f) of *Taxus cuspidata* in 2070. (a–c), RCP 2.6; (d–f), RCP 8.5. Red and green indicate high and low genetic offset, respectively. The details of the genetic offset values were showed in Table S9.

**FIGURE 4:**
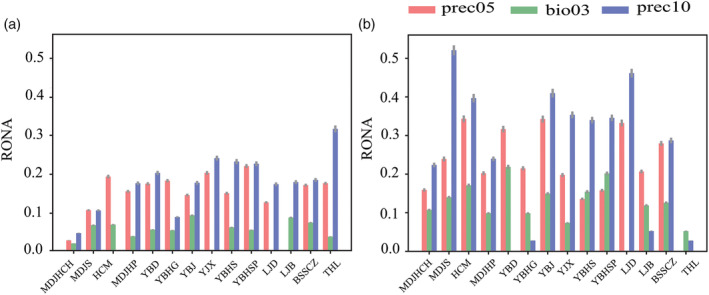
RONA of *Taxus cuspidata* under RCP 2.6 (a) and RCP 8.5 (b) prediction models for 2070. Bars represent weighted means (by *R* ^2^ value), and grey error bars represent the standard error.

In the three GF models by all SNPs, *F*_ST_‐outlier and GEA‐outlier SNPs, the predicted turnover in allele frequencies across the landscape followed a southwest to northeast direction: northeastern populations are expected to have higher genetic offsets in the Changbai Mountains and adjacent areas in the future (Figure 3a–f), the degree of genetic offset is slightly higher under RCP 8.5 than that of RCP 2.6 (Table S9). The RONA revealed that the most represented environmental variables were prec05, bio03, and prec10 (Table S10). Under the combined effect of the three most represented climate variables, population THL showed the highest RONA value for RCP 2.6 (Figure 4). MDJS, HCM and YBJ populations showed the highest RONA value for RCP 8.5 in 2070 (Figure 4). The THL and MDJS populations had a lower adaptive potential for prec10 by 2070.

## 4 DISCUSSION

Our study integrated population genetics and landscape genomic methods to explore neutral and non‐neutral genetic variations in *T. cuspidata* populations in the Changbai Mountains. The neutral genetic variation dataset revealed high genetic diversity and low genetic differentiation, whereas non‐neutral genetic variation showed high genetic differentiation. GEAs revealed that the contribution of climate to genetic variation was greater than that of geography, and precipitation played an important role in the putative adaptive genetic variation in response to climate change. We found that populations in the northeast range will be more vulnerable to future climate change as suggested by putative adaptive genetic variation. Our study provides insight into how neutral and putative adaptive genetic variation interacts with the environment, which is essential for future conservation and management of natural populations.

### 4.1 High genetic diversity was maintained in present fragmented populations

We integrated the patterns of neutral and non‐neutral genetic variation in *T. cuspidata* using nSSRs and RAD‐seq datasets. In this study, high current genetic diversity and low genetic differentiation were observed across the extant geographical range in China. The present results are in agreement with previous *T. cuspidata* genetic studies by cpDNA fragments in Russian and South Korea (Kozyrenko et al., 2017), cpDNA and mtDNA fragments in China (Su et al., 2018), and were similar to that of *T. baccata* from Poland by microsatellites (Litkowiec et al., 2018). In addition, admixture analysis revealed substantial gene flow among the populations. For fragmented and isolated *T. cuspidata* populations, substantial gene flow may compensate for the genetic barrier caused by fragmented habitats (Browne & Karubian, 2018; Hu et al., 2008; Petit et al., 2005).

Theoretically, fragmentation of species usually has a negative effect on genetic diversity compared to intact populations (Nybom, 2004; Vranckx et al., 2012). One possibility for the high genetic diversity and gene flow might be the outcrossing nature of the species. *T. cuspidata* is a wind pollination and typical bird‐dispersed plant. Gene flow may be accomplished by both seeds and pollen, and pollen often disperses over longer distances than seeds (Jordano, 2017; Kremer et al., 2012). However, this seems to be difficult, particularly for long‐distance intervals among the studied populations. For yew, a multi‐model approach indicated that 95% of the seed and pollen dispersal distances were <109 m and 704 m, respectively (Chybicki & Oleksa, 2018). An alternative explanation for high genetic diversity is the delayed sexual maturity of the species. The unbalanced ratio of females to males of *T. cuspidata* (1:2.3) was found in this region (Long et al., 2021). The sex ratio in dioecious plants might significantly affect genetic diversity (Rosche et al., 2018), e.g., the dioecy may increase genetic diversity due to obligate outcrossing, which has been found in *Pherospheara hookeriana* (Worth et al., 2021). Isolation occurred mainly throughout the last few decades and was probably not long enough to impact current genetic diversity (Münzbergová et al., 2013; Rosche et al., 2018). Therefore, the short duration of fragmentation tends to maintain the standing variations in long‐lived plants, which may delay adverse effect in the progenies of *T. cuspidata* (Aguilar et al., 2008; Vranckx et al., 2012).

High genetic differentiation was also observed in the putative adaptive genetic variation, the high *F*_ST_ values may be due to the fixation of different alleles in the local populations. This might also be caused by natural selection in response to changing environments, and adaptive processes may contribute to high *F*_ST_ values. Therefore, natural populations of *T. cuspidata* primarily stem from intense selective pressure imposed by environmental factors.

### 4.2 Signature of natural selection in *T. cuspidata*

Outlier detection test is an effective approach to identify the presence of putatively adaptive genetic variation. Although outlier detection tests can produce false outliers due to confounding factors (Schoville et al., 2012), tests with various demographic hypotheses can be utilized and compared to solve this issue, and common loci detected in consensus are more likely to be actual targets of selection (Ahrens et al., 2018). In this study, we applied three outlier methods to detect different sets of outlier SNPs that showed signatures of selection. The proportion of *F*_ST_ outlier (0.4%) aligns closely with the findings in other conifers, such as *Pinus taeda* (0.2%) (De La Torre et al., 2019), *Keteleeria davidiana* (0.2%) (Shih et al., 2018) and *Cupressus gigantea* (0.4%) (Yang et al., 2022). The proportion of GEA‐outlier SNPs (ranging from 1.7% to 1.8%) was similar to those of *T. baccata* (2.6% to 3.0%) (Mayol et al., 2020) and *Pinus albicaulis* (2.9%) (van Mantgem et al., 2023). In the present study, more GEA‐outlier SNPs associated with climatic variables were correlated with precipitation variables than temperature variables. These outlier SNPs of *T. cuspidata* suggested that signatures of selection may be involved in local adaptation processes in response to selective pressure. However, due to the short RAD reads and no annotated reference genome, we failed to further annotate any genes associated with putatively adaptive SNPs.

### 4.3 Genetic variation associated with adaptation to climate change

Precipitation and temperature are main climatic factors influencing plant's distribution and survival (Cuervo‐Alarcon et al., 2018). Temperature is a key factor influencing the growth and phenology of tree species (Schmiege et al., 2021; Vitasse et al., 2009). RDA and GF indicated that temperature was found to be associated with our nSSRs, neutral SNPs and all SNPs sets. An explanation to this temperature‐driven genetic variation is that temperature could affect the connection between populations (i.e., gene flow), which has been observed in yews in the northern Italy (Mercuri et al., 2013). Yews are also highly sensitive to extreme temperature changes, including *T. globosa* (Antúnez, 2021) and *T. baccata* (Mayol et al., 2020; Moir, 1999). Low temperatures can encourage the rooting of yews during the early growth stage (Muñoz‐Gutiérrez et al., 2009). Therefore, the maximum temperature of warmest month (bio05) and the mean temperature of driest quarter (bio09) might affect the growth and distribution of *T. cuspidata*.

We also found that precipitation was an important variable associated with putative adaptive genetic variation. Water availability may be an important selection factor for yew species, especially during the last interglacial period, as indicated by the association study in the common yew, *T. baccata* (Mayol et al., 2020). A previous study using ecological niche modeling has also found that the best suitable distribution time for *T. cuspidata* is during the last glacial maximum and the most restricted distribution time for the species is during the last interglacial period (c. 130 ka), suggesting the cold‐tolerant and wet‐sensitive features of the species (Su et al., 2018). Therefore, water availability will be a challenge for *T. cuspidata* when facing the changing environments, as suggested by other yew species (Linares, 2013).

### 4.4 Species vulnerability and conservation strategies

Assessing the vulnerability of species to climate change can provide insight into the potential risk of species persistence in future climate scenarios (Capblancq et al., 2020; Feng & Du, 2022). Genetic prediction of adaptation to future climate indicated that populations (e.g., MDJHP, YJX and MDJS) in the northeast area (Heilongjiang and Jilin Provinces) exhibited a higher risk of genetic maladaptation than other populations. These populations showed low potential to adapt to changing environments in small or isolated habitats.

For GF genetic offset, the predictive power of genomic offset estimates on fitness effects is increasingly being assessed through experimental and simulation studies, showing promising results (Láruson et al., 2022). The GF offset results in our study differed not much between the two future climate scenarios. An explanation for the phenomenon is that *T. cuspidata* has a long lifecycle with long generation intervals, in which the rates of emergence and spread of novel adaptive alleles in populations through de novo mutations are likely to be too slow to respond to climate change.

For all SNPs and putatively adaptive SNPs, northeastern populations showed higher genetic offsets and vulnerabilities than the other populations. Su et al. (2018) found that ecological niche modeling showed a contraction trend in the distribution of *T. cuspidata* by 2070, with northeastern populations located in a contracting area. Láruson et al. (2022) showed that higher genetic offset may be caused by genetics drift at small population rather than a selection‐driven response. Negative associations between GF offset and population size have also been found in previous bird studies (Bay et al., 2018; Ruegg et al., 2018). Therefore, the high genetic offset of *T. cuspidata* may also be caused by natural selection and/or small population size.

Based on our study and previous reports, we came to the conclusion that *T. cuspidata* is at risk in China with number of challenges, such as fragmentation and habitat loss, climate change, disturbance and the unbalanced ratio of females to males (Long et al., 2021; Su et al., 2018; Wang et al., 2019). Our study specifically recommends adopting in situ conservation strategies for THL, LJD and YBHSP populations with high adaptive genetic diversity but vulnerable in the future. In addition, populations displaying low adaptive variation, including MDJHCH, MDJHP and YBJ, could benefit from ex situ conservation strategies. Furthermore, for small or isolated populations characterized by limited genetic diversity and high vulnerability (e.g., BSSCZ, YBJ), genetic rescue and assisted gene flow techniques should be considered to facilitate their adaptation to climate change. Previous studies have demonstrated the effectiveness of such approaches (Aitken & Bemmels, 2016; Aitken & Whitlock, 2013; Whiteley et al., 2015).

It is worth noting that our study represents a pioneering effort to integrate neutral and adaptive genetic variation to the conservation of a threatened tree species. However, to gain deeper insights into the molecular mechanisms underlying the threats faced by *T. cuspidata*, future research endeavors should include comprehensive investigations encompassing whole‐genome or transcriptome sequencing, along with the inclusion of phenotypic and fine‐scale environmental data.

## CONFLICT OF INTEREST STATEMENT

The authors declare no conflict of interest.

## Supporting information


Figure S1.

Table S1.

## Data Availability

Data for this study are available at 10.6084/m9.figshare.23515398.

## References

[eva13686-bib-0001] Aguilar, R. , Quesada, M. , Ashworth, L. , Herrerias‐Diego, Y. V. O. N. N. E. , & Lobo, J. (2008). Genetic consequences of habitat fragmentation in plant populations: Susceptible signals in plant traits and methodological approaches. Molecular Ecology, 17(24), 5177–5188. 10.1111/j.1365-294X.2008.03971.x 19120995

[eva13686-bib-0002] Ahrens, C. W. , Rymer, P. D. , Stow, A. , Bragg, J. , Dillon, S. , Umbers, K. D. , & Dudaniec, R. Y. (2018). The search for loci under selection: Trends, biases and progress. Molecular Ecology, 27(6), 1342–1356. 10.1111/mec.14549 29524276

[eva13686-bib-0003] Aitken, S. N. , & Bemmels, J. B. (2016). Time to get moving: Assisted gene flow of forest trees. Evolutionary Applications, 9(1), 271–290. 10.1111/eva.12293 27087852 PMC4780373

[eva13686-bib-0004] Aitken, S. N. , & Whitlock, M. C. (2013). Assisted gene flow to facilitate local adaptation to climate change. Annual Review of Ecology, Evolution, and Systematics, 44, 367–388. 10.1146/annurev-ecolsys-110512-135747

[eva13686-bib-0005] Alexander, D. H. , & Lange, K. (2011). Enhancements to the ADMIXTURE algorithm for individual ancestry estimation. BMC Bioinformatics, 12, 1–6. 10.1186/1471-2105-12-246 21682921 PMC3146885

[eva13686-bib-0006] Alexander, D. H. , Novembre, J. , & Lange, K. (2009). Fast model‐based estimation of ancestry in unrelated individuals. Genome Research, 19(9), 1655–1664. 10.1101/gr.094052.109 19648217 PMC2752134

[eva13686-bib-0007] Andrews, S. (2010). FastQC: A quality control tool for high throughput sequence data. http://www.bioinformatics.babraham.ac.uk/projects/fastqc/

[eva13686-bib-0008] Antúnez, P. (2021). Influence of physiography, soil and climate on *Taxus globosa* . Nordic Journal of Botany, 39(3), e03058. 10.1111/njb.03241

[eva13686-bib-0009] Bay, R. A. , Harrigan, R. J. , Underwood, V. L. , Gibbs, H. L. , Smith, T. B. , & Ruegg, K. (2018). Genomic signals of selection predict climate‐driven population declines in a migratory bird. Science, 359(6371), 83–86. 10.1126/science.aan438 29302012

[eva13686-bib-0010] Bolger, A. M. , Lohse, M. , & Usadel, B. (2014). Trimmomatic: A flexible trimmer for Illumina sequence data. Bioinformatics, 30, 2114–2120. 10.1093/bioinformatics/btu170 24695404 PMC4103590

[eva13686-bib-0011] Bonan, G. B. (2008). Forests and climate change: Forcings, feedbacks, and the climate benefits of forests. Science, 320(5882), 1444–1449. 10.1126/science.1155121 18556546

[eva13686-bib-0012] Borrell, J. S. , Zohren, J. , Nichols, R. A. , & Buggs, R. J. (2020). Genomic assessment of local adaptation in dwarf birch to inform assisted gene flow. Evolutionary Applications, 13(1), 161–175. 10.1111/eva.12883 31892950 PMC6935589

[eva13686-bib-0013] Browne, L. , & Karubian, J. (2018). Habitat loss and fragmentation reduce effective gene flow by disrupting seed dispersal in a neotropical palm. Molecular Ecology, 27(15), 3055–3069. 10.1111/mec.14765 29900620

[eva13686-bib-0014] Capblancq, T. , Fitzpatrick, M. C. , Bay, R. A. , Exposito‐Alonso, M. , & Keller, S. R. (2020). Genomic prediction of (mal) adaptation across current and future climatic landscapes. Annual Review of Ecology, Evolution, and Systematics, 51, 245–269. 10.1146/annurev-ecolsys-020720-042553

[eva13686-bib-0015] Catchen, J. , Hohenlohe, P. A. , Bassham, S. , Amores, A. , & Cresko, W. A. (2013). Stacks: An analysis tool set for population genomics. Molecular Ecology, 22(11), 3124–3140. 10.1111/mec.12354 23701397 PMC3936987

[eva13686-bib-0016] Catchen, J. M. , Amores, A. , Hohenlohe, P. , Cresko, W. , & Postlethwait, J. H. (2011). Stacks: Building and genotyping loci de novo from short‐read sequences. G3: Genes, Genomes, Genetics, 1(3), 171–182. 10.1534/g3.111.000240 22384329 PMC3276136

[eva13686-bib-0017] Cheng, B. B. , Zheng, Y. Q. , & Sun, Q. W. (2015). Genetic diversity and population structure of *Taxus cuspidata* in the Changbai Mountains assessed by chloroplast DNA sequences and microsatellite markers. Biochemical Systematics and Ecology, 63, 157–164. 10.1016/j.bse.2015.10.009

[eva13686-bib-0018] Chung, M. G. , Oh, G. S. , & Chung, J. M. (1999). Allozyme variation in Korean populations of *Taxus cuspidata* (Taxaceae). Scandinavian Journal of Forest Research, 14(2), 103–110. 10.1080/02827589950152827

[eva13686-bib-0019] Chung, M. Y. , Merilä, J. , Li, J. , Mao, K. , López‐Pujol, J. , Tsumura, Y. , & Chung, M. G. (2023). Neutral and adaptive genetic diversity in plants: An overview. Frontiers in Ecology and Evolution, 11, 1116814. 10.3389/fevo.2023.1116814

[eva13686-bib-0020] Chybicki, I. J. , & Oleksa, A. (2018). Seed and pollen gene dispersal in *Taxus baccata*, a dioecious conifer in the face of strong population fragmentation. Annals of Botany, 122(3), 409–421. 10.1093/aob/mcy081 29873697 PMC6311948

[eva13686-bib-0021] Cuervo‐Alarcon, L. , Arend, M. , Müller, M. , Sperisen, C. , Finkeldey, R. , & Krutovsky, K. V. (2018). Genetic variation and signatures of natural selection in populations of European beech (*Fagus sylvatica* L.) along precipitation gradients. Tree Genetics & Genomes, 14, 1–21. 10.1007/s11295-018-1297-2

[eva13686-bib-0022] Danecek, P. , Auton, A. , Abecasis, G. , Albers, C. A. , Banks, E. , DePristo, M. A. , Handsaker, R. E. , Lunter, G. , Marth, G. T. , Sherry, S. T. , McVean, G. , & Durbin, R . (2011). The variant call format and VCFtools. Bioinformatics, 27(15), 2156–2158. 10.1093/bioinformatics/btr330 21653522 PMC3137218

[eva13686-bib-0023] De La Torre, A. R. , Wilhite, B. , & Neale, D. B. (2019). Environmental genome‐wide association reveals climate adaptation is shaped by subtle to moderate allele frequency shifts in loblolly pine. Genome Biology and Evolution, 11(10), 2976–2989. 10.1093/gbe/evz220 31599932 PMC6821164

[eva13686-bib-0024] de Sousa, V. A. , Reeves, P. A. , Reilley, A. , de Aguiar, A. V. , Stefenon, V. M. , & Richards, C. M. (2020). Genetic diversity and biogeographic determinants of population structure in *Araucaria angustifolia* (Bert.) O. Ktze. Conservation Genetics, 21(2), 217–229. 10.1007/s10592-019-01242-9

[eva13686-bib-0025] Du, F. K. , Hou, M. , Wang, W. , Mao, K. , & Hampe, A. (2017). Phylogeography of *Quercus aquifolioides* provides novel insights into the Neogene history of a major global hotspot of plant diversity in south‐west China. Journal of Biogeography, 44(2), 294–307. 10.1111/jbi.12836

[eva13686-bib-0026] Du, F. K. , Wang, T. , Wang, Y. , Ueno, S. , & de Lafontaine, G. (2020). Contrasted patterns of local adaptation to climate change across the range of an evergreen oak, *Quercus aquifolioides* . Evolutionary Applications, 13(9), 2377–2391. 10.1111/eva.13030 33005228 PMC7513717

[eva13686-bib-0027] Dubreuil, M. , Sebastiani, F. , Mayol, M. , González‐Martínez, S. C. , Riba, M. , & Vendramin, G. G. (2008). Isolation and characterization of polymorphic nuclear microsatellite loci in *Taxus baccata* L. Conservation Genetics, 9, 1665–1668. 10.1007/s10592-008-9515-3

[eva13686-bib-0028] Earl, D. A. , & VonHoldt, B. M. (2012). STRUCTURE HARVESTER: A website and program for visualizing STRUCTURE output and implementing the Evanno method. Conservation Genetics Resources, 4, 359–361. 10.1007/s12686-011-9548-7

[eva13686-bib-0029] Ellis, N. , Smith, S. J. , & Pitcher, C. R. (2012). Gradient forests: Calculating importance gradients on physical predictors. Ecology, 93(1), 156–168. 10.1890/11-0252.1 22486096

[eva13686-bib-0030] Evanno, G. , Regnaut, S. , & Goudet, J. (2005). Detecting the number of clusters of individuals using the software STRUCTURE: A simulation study. Molecular Ecology, 14(8), 2611–2620. 10.1111/j.1365-294X.2005.02553.x 15969739

[eva13686-bib-0031] Excoffier, L. , & Lischer, H. E. (2010). Arlequin suite ver 3.5: A new series of programs to perform population genetics analyses under Linux and windows. Molecular Ecology Resources, 10(3), 564–567. 10.1111/j.1755-0998.2010.02847.x 21565059

[eva13686-bib-0032] Feng, L. , & Du, F. K. (2022). Landscape genomics in tree conservation under a changing environment. Frontiers in Plant Science, 13(822), 217. 10.3389/fpls.2022.822217 PMC8908315 35283901

[eva13686-bib-0033] Fick, S. E. , & Hijmans, R. J. (2017). WorldClim 2: New 1‐km spatial resolution climate surfaces for global land areas. International Journal of Climatology, 37(12), 4302–4315. 10.1002/joc.5086

[eva13686-bib-0034] Fitzpatrick, M. C. , & Keller, S. R. (2015). Ecological genomics meets community‐level modelling of biodiversity: Mapping the genomic landscape of current and future environmental adaptation. Ecology Letters, 18(1), 1–16. 10.1111/ele.12376 25270536

[eva13686-bib-0035] Foll, M. , & Gaggiotti, O. (2008). A genome‐scan method to identify selected loci appropriate for both dominant and codominant markers: A Bayesian perspective. Genetics, 180(2), 977–993. 10.1534/genetics.108.092221 18780740 PMC2567396

[eva13686-bib-0036] Fox, J. , & Weisberg, S. (2019). An R companion to applied regression (Third ed.). Sage. https://socialsciences.mcmaster.ca/jfox/Books/Companion/

[eva13686-bib-0037] Frankham, R. (2005). Conservation biology: Ecosystem recovery enhanced by genotypic diversity. Heredity, 95(3), 183. 10.1038/sj.hdy.6800706 16049423

[eva13686-bib-0038] Frichot, E. , & François, O. (2015). LEA: An R package for landscape and ecological association studies. Methods in Ecology and Evolution, 6(8), 925–929. 10.1111/2041-210x.12382

[eva13686-bib-0039] Frichot, E. , Schoville, S. D. , Bouchard, G. , & François, O. (2013). Testing for associations between loci and environmental gradients using latent factor mixed models. Molecular Biology and Evolution, 30, 1687–1699. 10.1093/molbev/mst063 23543094 PMC3684853

[eva13686-bib-0040] Gitzendanner, M. A. , Weekley, C. W. , Germain‐Aubrey, C. C. , Soltis, D. E. , & Soltis, P. S. (2012). Microsatellite evidence for high clonality and limited genetic diversity in *Ziziphus celata* (Rhamnaceae), an endangered, self‐incompatible shrub endemic to the Lake Wales ridge, Florida, USA. Conservation Genetics, 13, 223–234. 10.1007/s10592-011-0287-9

[eva13686-bib-0041] González, A. V. , Gómez‐Silva, V. , Ramírez, M. J. , & Fontúrbel, F. E. (2020). Meta‐analysis of the differential effects of habitat fragmentation and degradation on plant genetic diversity. Conservation Biology, 34(3), 711–720. 10.1111/cobi.13422 31605401

[eva13686-bib-0042] Günther, T. , & Coop, G. (2013). Robust identification of local adaptation from allele frequencies. Genetics, 195(1), 205–220. 10.1534/genetics.113.152462 23821598 PMC3761302

[eva13686-bib-0043] Hoffmann, A. A. , & Willi, Y. (2008). Detecting genetic responses to environmental change. Nature Reviews Genetics, 9(6), 421–432. 10.1038/nrg2339 18463665

[eva13686-bib-0044] Holderegger, R. , Kamm, U. , & Gugerli, F. (2006). Adaptive vs. neutral genetic diversity: Implications for landscape genetics. Landscape Ecology, 21, 797–807. 10.1007/s10980-005-5245-9

[eva13686-bib-0045] Hu, L. J. , Uchiyama, K. , Shen, H. L. , Saito, Y. , Tsuda, Y. , & Ide, Y. (2008). Nuclear DNA microsatellites reveal genetic variation but a lack of phylogeographical structure in an endangered species, *Fraxinus mandshurica*, across north‐east China. Annals of Botany, 102(2), 195–205. 10.1093/aob/mcn074 18477559 PMC2712365

[eva13686-bib-0046] IPCC . (2014). Climate change 2014: Synthesis report. Contribution of working groups I. II and III to the fifth assessment report of the intergovernmental panel on climate change 151(10.1017). IPCC.

[eva13686-bib-0047] Jakobsson, M. , & Rosenberg, N. A. (2007). CLUMPP: A cluster matching and permutation program for dealing with label switching and multimodality in analysis of population structure. Bioinformatics, 23(14), 1801–1806. 10.1093/bioinformatics/btm233 17485429

[eva13686-bib-0048] Jia, K. H. , Zhao, W. , Maier, P. A. , Hu, X. G. , Jin, Y. , Zhou, S. S. , … Mao, J. F. (2020). Landscape genomics predicts climate change‐related genetic offset for the widespread *Platycladus orientalis* (Cupressaceae). Evolutionary Applications, 13(4), 665–676. 10.1111/eva.12891 32211059 PMC7086053

[eva13686-bib-0049] Jombart, T. , & Ahmed, I. (2011). Adegenet 1.3–1: New tools for the analysis of genome‐wide SNP data. Bioinformatics, 27(21), 3070–3071. 10.1093/bioinformatics/btr521 21926124 PMC3198581

[eva13686-bib-0050] Jordano, P. (2017). What is long‐distance dispersal? And a taxonomy of dispersal events. Journal of Ecology, 105(1), 75–84. 10.1111/1365-2745.12690

[eva13686-bib-0051] Katsuki, T. , & Luscombe, D. (2013). *Taxus cuspidata*. The IUCN red list of threatened species. 2013: E.T42549A2987373. 10.2305/IUCN.UK.2013-1

[eva13686-bib-0052] Kitamura, S. , & Murata, G. (1987). Colored illustrations of woody plants of Japan (Vol. II, p. 545). Hoikusha Publ. Co., Ltd.

[eva13686-bib-0053] Kondo, T. (2016). Development of polymorphic microsatellite markers for Japanese yew, *Taxus cuspidata*, and *T. Cuspidata* var. *nana* (Taxaceae). Applications in Plant Sciences, 4(7), 1600020. 10.3732/apps.1600020 PMC4948900 27437172

[eva13686-bib-0054] Kozyrenko, M. M. , Artyukova, E. V. , & Chubar, E. A. (2017). Genetic diversity and population structure of *Taxus cuspidata* Sieb. Et Zucc. Ex Endl. (Taxaceae) in Russia according to data of the nucleotide polymorphism of intergenic spacers of the chloroplast genome. Russian Journal of Genetics, 53, 865–874. 10.1134/S1022795417070079

[eva13686-bib-0055] Kremer, A. , Ronce, O. , Robledo‐Arnuncio, J. J. , Guillaume, F. , Bohrer, G. , Nathan, R. , … Schueler, S. (2012). Long‐distance gene flow and adaptation of forest trees to rapid climate change. Ecology Letters, 15(4), 378–392. 10.1111/j.1461-0248.2012.01746.x 22372546 PMC3490371

[eva13686-bib-0056] Laikre, L. , Allendorf, F. W. , Aroner, L. C. , Baker, C. S. , Gregovich, D. P. , Hansen, M. M. , … Waples, R. S. (2010). Neglect of genetic diversity in implementation of the convention of biological diversity. Conservation Biology, 24(1), 86–88. 10.1111/j.1523-1739.2009.01425.x 20028412

[eva13686-bib-0057] Láruson, Á. J. , Fitzpatrick, M. C. , Keller, S. R. , Haller, B. C. , & Lotterhos, K. E. (2022). Seeing the forest for the trees: Assessing genetic offset predictions from gradient forest. Evolutionary Applications, 15(3), 403–416. 10.1111/eva.13354 35386401 PMC8965365

[eva13686-bib-0058] Linares, J. C. (2013). Shifting limiting factors for population dynamics and conservation status of the endangered English yew (*Taxus baccata* L., Taxaceae). Forest Ecology and Management, 291, 119–127. 10.1016/j.foreco.2012.11.009

[eva13686-bib-0059] Litkowiec, M. , Lewandowski, A. , & Wachowiak, W. (2018). Genetic variation in *Taxus baccata* L.: A case study supporting Poland's protection and restoration program. Forest Ecology and Management, 409, 148–160. 10.1016/j.foreco.2017.11.026

[eva13686-bib-0060] Long, T. , Wu, X. , Wang, Y. , Chen, J. , Xu, C. , Li, J. , … Zang, R. (2021). The population status and threats of *Taxus cuspidata*, a plant species with extremely small populations in China. Global Ecology and Conservation, 26, e01495. 10.1016/j.gecco.2021.e01495

[eva13686-bib-0061] Lowe, A. J. , Boshier, D. , Ward, M. , Bacles, C. F. E. , & Navarro, C. (2005). Genetic resource impacts of habitat loss and degradation; reconciling empirical evidence and predicted theory for neotropical trees. Heredity, 95(4), 255–273. 10.1038/sj.hdy.6800725 16094300

[eva13686-bib-0062] Mackay, J. , Dean, J. F. , Plomion, C. , Peterson, D. G. , Cánovas, F. M. , Pavy, N. , … Cervera, M. T. (2012). Towards decoding the conifer giga‐genome. Plant Molecular Biology, 80, 555–569. 10.1007/s11103-012-9961-7 22960864

[eva13686-bib-0063] Marquaridt, D. W. (1970). Generalized inverses, ridge regression, biased linear estimation, and nonlinear estimation. Technometrics, 12(3), 591–612. 10.1080/00401706.1970.10488699

[eva13686-bib-0064] Mayol, M. , Riba, M. , Cavers, S. , Grivet, D. , Vincenot, L. , Cattonaro, F. , … González‐Martínez, S. C. (2020). A multiscale approach to detect selection in nonmodel tree species: Widespread adaptation despite population decline in *Taxus baccata* L. Evolutionary Applications, 13(1), 143–160. 10.1111/eva.12838 31892949 PMC6935595

[eva13686-bib-0065] Mercuri, A. M. , Torri, P. , Casini, E. , & Olmi, L. (2013). Climate warming and the decline of Taxus airborne pollen in urban pollen rain (Emilia Romagna, northern Italy). Plant Biology, 15, 70–82. 10.1111/j.1438-8677.2012.00624.x 22776105

[eva13686-bib-0066] Miao, Y. C. , Zhang, Z. J. , & Su, J. R. (2016). Low genetic diversity in the endangered *Taxus yunnanensis* following a population bottleneck, a low effective population size and increased inbreeding. Silvae Genetica, 65, 59–66. 10.1515/sg-2016-0008

[eva13686-bib-0067] Moir, A. K. (1999). The dendrochronological potential of modern yew (*Taxus baccata*) with special reference to yew from Hampton court palace. UK. The New Phytologist, 144(3), 479–488. 10.1046/j.1469-8137.1999.00545.x 33862863

[eva13686-bib-0068] Muñoz‐Gutiérrez, L. , Vargas‐Hernández, J. J. , López‐Upton, J. , & Soto‐Hernández, M. (2009). Effect of cutting age and substrate temperature on rooting of *Taxus globosa* . New Forests, 38, 187–196. 10.1007/s11056-009-9139-6

[eva13686-bib-0069] Münzbergová, Z. , Cousins, S. A. , Herben, T. , Plačková, I. , Mildén, M. , & Ehrlén, J. (2013). Historical habitat connectivity affects current genetic structure in a grassland species. Plant Biology, 15(1), 195–202. 10.1111/j.1438-8677.2012.00601.x 22646655

[eva13686-bib-0070] Naimi, B. , Hamm, N. A. , Groen, T. A. , Skidmore, A. K. , & Toxopeus, A. G. (2014). Where is positional uncertainty a problem for species distribution modelling? Ecography, 37(2), 191–203. 10.1111/j.1600-0587.2013.00205.x

[eva13686-bib-0071] Neale, D. B. , & Kremer, A. (2011). Forest tree genomics: Growing resources and applications. Nature Reviews Genetics, 12(2), 111–122. 10.1038/nrg2931 21245829

[eva13686-bib-0072] Nielsen, E. S. , Hanson, J. O. , Carvalho, S. B. , Beger, M. , Henriques, R. , Kershaw, F. , & Von der Heyden, S. (2022). Molecular ecology meets systematic conservation planning. Trends in Ecology & Evolution, 38, 143–155. 10.1016/j.tree.2022.09.006 36210287

[eva13686-bib-0073] Nybom, H. (2004). Comparison of different nuclear DNA markers for estimating intraspecific genetic diversity in plants. Molecular Ecology, 13(5), 1143–1155. 10.1111/j.1365-294X.2004.02141.x 15078452

[eva13686-bib-0074] O'Brien, D. , Laikre, L. , Hoban, S. , Bruford, M. W. , Ekblom, R. , Fischer, M. C. , … MacDonald, A. J. (2022). Bringing together approaches to reporting on within species genetic diversity. Journal of Applied Ecology, 59(9), 2227–2233. 10.1111/1365-2664.14

[eva13686-bib-0075] Oksanen, J. , Blanchet, F. G. , Friendly, M. , Kindt, R. , Legendre, P. , McGlinn, D. , … Imports, M. A. S. S. (2019). Package ‘vegan’. Community Ecology Package, Version 2.9. https://CRAN.R‐project.org/package=vegan

[eva13686-bib-0076] Peakall, R. O. D. , & Smouse, P. E. (2006). GENALEX 6: Genetic analysis in excel. Population genetic software for teaching and research. Molecular Ecology Notes, 6(1), 288–295. 10.1093/bioinformatics/bts460 PMC3463245 22820204

[eva13686-bib-0077] Peterson, B. K. , Weber, J. N. , Kay, E. H. , Fisher, H. S. , & Hoekstra, H. E. (2012). Double digest RADseq: An inexpensive method for de novo SNP discovery and genotyping in model and non‐model species. PLoS One, 7(5), e37135. 10.1371/journal.pone.0037135 22675423 PMC3365034

[eva13686-bib-0078] Petit, J. R. , Duminil, J. , Fineschi, S. , Hampe, A. , Salvini, D. , & Ven‐dramin, G. G. (2005). Comparative organization of chloroplast, mitochondrial and nuclear diversity in plant populations. Molecular Ecology, 14, 689–701. 10.1111/j.1365-294X.2004.02410.x 15723661

[eva13686-bib-0079] Petit, R. J. , & Hampe, A. (2006). Some evolutionary consequences of being a tree. Annual Review of Ecology, Evolution, and Systematics, 37, 187–214. 10.1146/annurev.ecolsys.37.091305.1102

[eva13686-bib-0080] Pina‐Martins, F. , Baptista, J. , Pappas, G., Jr. , & Paulo, O. S. (2019). New insights into adaptation and population structure of cork oak using genotyping by sequencing. Global Change Biology, 25(1), 337–350. 10.1111/gcb.14497 30358018

[eva13686-bib-0081] Piotti, A. (2009). The genetic consequences of habitat fragmentation: The case of forests. iForest – Biogeosciences and Forestry, 2(3), 75–76. 10.3832/ifor0496-002

[eva13686-bib-0082] Pritchard, J. K. , Stephens, M. , & Donnelly, P. (2000). Inference of population structure using multilocus genotype data. Genetics, 155(2), 945–959. 10.1093/genetics/155.2.945 10835412 PMC1461096

[eva13686-bib-0083] Puebla, O. , Bermingham, E. , & McMillan, W. O. (2014). Genomic atolls of differentiation in coral reef fishes (*Hypoplectrus* spp., *Serranidae*). Molecular Ecology, 23(21), 5291–5303. 10.1111/mec.12926 25231270

[eva13686-bib-0084] Purcell, S. , Neale, B. , Todd‐Brown, K. , Thomas, L. , Ferreira, M. A. , Bender, D. , … Sham, P. C. (2007). PLINK: A tool set for whole‐genome association and population‐based linkage analyses. The American Journal of Human Genetics, 81(3), 559–575. 10.1086/519795 17701901 PMC1950838

[eva13686-bib-0085] Pykälä, J. (2019). Habitat loss and deterioration explain the disappearance of populations of threatened vascular plants, bryophytes and lichens in a hemiboreal landscape. Global Ecology and Conservation, 18, e00610. 10.1016/j.gecco.2019.e00610

[eva13686-bib-0086] R Core Team . (2019). R: A language and environment for statistical computing. R Foundation for Statistical Computing. https://www.R‐project.org/

[eva13686-bib-0087] Rands, M. R. , Adams, W. M. , Bennun, L. , Butchart, S. H. , Clements, A. , Coomes, D. , … Vira, B. (2010). Biodiversity conservation: Challenges beyond 2010. Science, 329(5997), 1298–1303. 10.1126/science.1189138 20829476

[eva13686-bib-0088] Rellstab, C. , Zoller, S. , Walthert, L. , Lesur, I. , Pluess, A. R. , Graf, R. , … Gugerli, F. (2016). Signatures of local adaptation in candidate genes of oaks (*Quercus* spp.) with respect to present and future climatic conditions. Molecular Ecology, 25(23), 5907–5924. 10.1111/mec.13889 27759957

[eva13686-bib-0089] Rosche, C. , Schrieber, K. , Lachmuth, S. , Durka, W. , Hirsch, H. , Wagner, V. , … Hensen, I. (2018). Sex ratio rather than population size affects genetic diversity in *Antennaria dioica* . Plant Biology, 20(4), 789–796. 10.1111/plb.12716 29521023

[eva13686-bib-0090] Rosenberg, N. A. (2004). DISTRUCT: A program for the graphical display of population structure. Molecular Ecology Notes, 4(1), 137–138. 10.1046/j.1471-8286.2003.00566.x

[eva13686-bib-0091] Ruegg, K. , Bay, R. A. , Anderson, E. C. , Saracco, J. F. , Harrigan, R. J. , Whitfield, M. , … Smith, T. B. (2018). Ecological genomics predicts climate vulnerability in an endangered southwestern songbird. Ecology Letters, 21(7), 1085–1096. 10.1111/ele.12977 29745027

[eva13686-bib-0092] Schmiege, S. C. , Buckley, B. M. , Stevenson, D. W. , Heskel, M. A. , Cuong, T. Q. , Nam, L. C. , & Griffin, K. L. (2021). Respiratory temperature responses of tropical conifers differ with leaf morphology. Functional Ecology, 35(7), 1408–1423. 10.1111/1365-2435.13814

[eva13686-bib-0093] Schoville, S. D. , Bonin, A. , François, O. , Lobreaux, S. , Melodelima, C. , & Manel, S. (2012). Adaptive genetic variation on the landscape: Methods and cases. Annual Review of Ecology, Evolution, and Systematics, 43, 23–43. 10.1146/annurev-ecolsys-110411-160248

[eva13686-bib-0094] Shih, K. M. , Chang, C. T. , Chung, J. D. , Chiang, Y. C. , & Hwang, S. Y. (2018). Adaptive genetic divergence despite significant isolation‐by‐distance in populations of Taiwan cow‐tail fir (*Keteleeria davidiana* var. *formosana*). Frontiers Plant Science, 9, 92. 10.3389/fpls.2018.00092 PMC5799944 29449860

[eva13686-bib-0095] Sork, V. L. , Aitken, S. N. , Dyer, R. J. , Eckert, A. J. , Legendre, P. , & Neale, D. B. (2013). Putting the landscape into the genomics of trees: Approaches for understanding local adaptation and population responses to changing climate. Tree Genetics & Genomes, 9, 901–911. 10.1007/s11295-013-0596-x

[eva13686-bib-0096] Su, J. , Yan, Y. , Song, J. , Li, J. , Mao, J. , Wang, N. , … Du, F. K. (2018). Recent fragmentation may not alter genetic patterns in endangered long‐lived species: Evidence from *Taxus cuspidata* . Frontiers in Plant Science, 9, 1571. 10.3389/fpls.2018.01571 30429863 PMC6220038

[eva13686-bib-0097] Tang, S. , Dai, W. , Li, M. , Zhang, Y. , Geng, Y. , Tang, L. , & Zhong, Y. (2008). Genetic diversity of relictual and endangered plant *Abies ziyuanensis* (Pinaceae) revealed by AFLP and SSR markers. Genetica, 133, 21–30. 10.1007/s10709-007-9178-x 17661154

[eva13686-bib-0098] van Mantgem, P. J. , Milano, E. R. , Dudney, J. , Nesmith, J. C. , Vandergast, A. G. , & Zald, H. S. (2023). Growth, drought response, and climate‐associated genomic structure in whitebark pine in the Sierra Nevada of California. Ecology and Evolution, 13(5), e10072. 10.1002/ece3.10072 37206686 PMC10191741

[eva13686-bib-0099] Vitasse, Y. , Delzon, S. , Bresson, C. C. , Michalet, R. , & Kremer, A. (2009). Altitudinal differentiation in growth and phenology among populations of temperate‐zone tree species growing in a common garden. Canadian Journal of Forest Research, 39(7), 1259–1269. 10.1139/X09-054

[eva13686-bib-0100] Vranckx, G. U. Y. , Jacquemyn, H. , Muys, B. , & Honnay, O. (2012). Meta‐analysis of susceptibility of woody plants to loss of genetic diversity through habitat fragmentation. Conservation Biology, 26(2), 228–237. 10.1111/j.1523-1739.2011.01778.x 22044646

[eva13686-bib-0101] Wade, E. M. , Nadarajan, J. , Yang, X. , Ballesteros, D. , Sun, W. , & Pritchard, H. W. (2016). Plant species with extremely small populations (PSESP) in China: A seed and spore biology perspective. Plant Diversity, 38(5), 209–220. 10.1016/j.pld.2016.09.002 30159468 PMC6112217

[eva13686-bib-0102] Wang, H. W. , & Ge, S. (2006). Phylogeography of the endangered *Cathaya argyrophylla* (Pinaceae) inferred from sequence variation of mitochondrial and nuclear DNA. Molecular Ecology, 15(13), 4109–4122. 10.1111/j.1365-294X.2006.03086.x 17054506

[eva13686-bib-0103] Wang, J. , Wang, Y. , Feng, J. , Chen, C. , Chen, J. , Long, T. , … Li, J. (2019). Differential responses to climate and land‐use changes in threatened Chinese *Taxus* species. Forests, 10(9), 766. 10.3390/f10090766

[eva13686-bib-0104] Wang, T. , Feng, L. , & Du, F. K. (2021). New approaches for ecological adaptation study: From population genetics to landscape genomics. Scientia Sinica Vitae, 51, 167–178. 10.1360/SSV-2020-0265

[eva13686-bib-0105] Whiteley, A. R. , Fitzpatrick, S. W. , Funk, W. C. , & Tallmon, D. A. (2015). Genetic rescue to the rescue. Trends in Ecology & Evolution, 30(1), 42–49. 10.1016/j.tree.2014.10.009 25435267

[eva13686-bib-0106] Worth, J. R. , Marthick, J. R. , Harrison, P. A. , Sakaguchi, S. , & Jordan, G. J. (2021). The palaeoendemic conifer *Pherosphaera hookeriana* (Podocarpaceae) exhibits high genetic diversity despite quaternary range contraction and post glacial bottlenecking. Conservation Genetics, 22, 307–321. 10.1007/s10592-021-01338-1

[eva13686-bib-0107] Yang, H. , Li, J. , Milne, R. I. , Tao, W. , Wang, Y. , Miao, J. , … Mao, K. (2022). Genomic insights into the genotype–environment mismatch and conservation units of a Qinghai–Tibet plateau endemic cypress under climate change. Evolutionary Applications, 15(6), 919–933. 10.1111/eva.13377 35782009 PMC9234613

[eva13686-bib-0108] Yang, J. , Cai, L. , Liu, D. , Chen, G. , Gratzfeld, J. , & Sun, W. (2020). China's conservation program on plant species with extremely small populations (PSESP): Progress and perspectives. Biological Conservation, 244(108), 535. 10.1016/j.biocon.2020.108535

[eva13686-bib-0109] Young, A. , Boyle, T. , & Brown, T. (1996). The population genetic consequences of habitat fragmentation for plants. Trends in Ecology & Evolution, 11(10), 413–418. 10.1016/0169-5347(96)10045-8 21237900

[eva13686-bib-0110] Yousefzadeh, H. , Rajaei, R. , Jasińska, A. , Walas, Ł. , Fragnière, Y. , & Kozlowski, G. (2018). Genetic diversity and differentiation of the riparian relict tree *Pterocarya fraxinifolia* (Juglandaceae) along altitudinal gradients in the Hyrcanian forest (Iran). Silva Fennica, 52(5), 10000. 10.14214/sf.10000

[eva13686-bib-0111] Zhao, W. , Sun, Y. Q. , Pan, J. , Sullivan, A. R. , Arnold, M. L. , Mao, J. F. , & Wang, X. R. (2020). Effects of landscapes and range expansion on population structure and local adaptation. New Phytologist, 228(1), 330–343. 10.1111/nph.16619 32323335

[eva13686-bib-0112] Zheng, X. , Levine, D. , Shen, J. , Gogarten, S. M. , Laurie, C. , & Weir, B. S. (2012). A high‐performance computing toolset for relatedness and principal component analysis of SNP data. Bioinformatics, 28(24), 3326–3328. 10.1093/bioinformatics/bts606 23060615 PMC3519454

