# Supplementary material for: Climate change vulnerability and conservation strategies for tertiary relict tree species: Insights from landscape genomics of Taxus cuspidata
Source: Evol Appl. 2024 Sep 4;17(9):e13686. doi: 10.1111/eva.13686 (PMC11375028; doi:10.1111/eva.13686)
Supplement: Supplementary file 1 — Figure S1. [file EVA-17-e13686-s002.docx]

**Supplementary Figures**


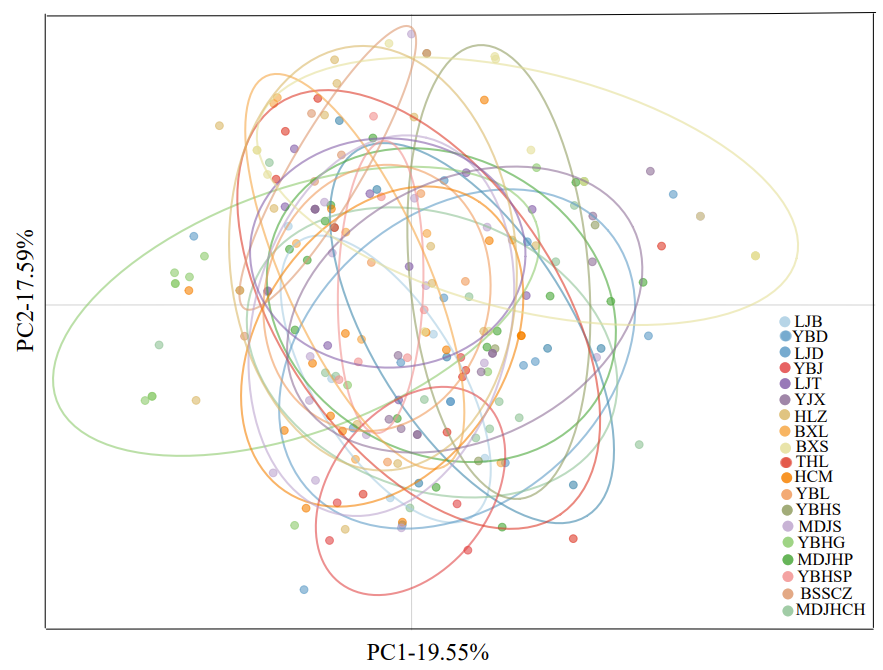


**Fig. S1.** Principal component analysis (PCA) of *T. cuspidata* by nSSRs dataset.

**
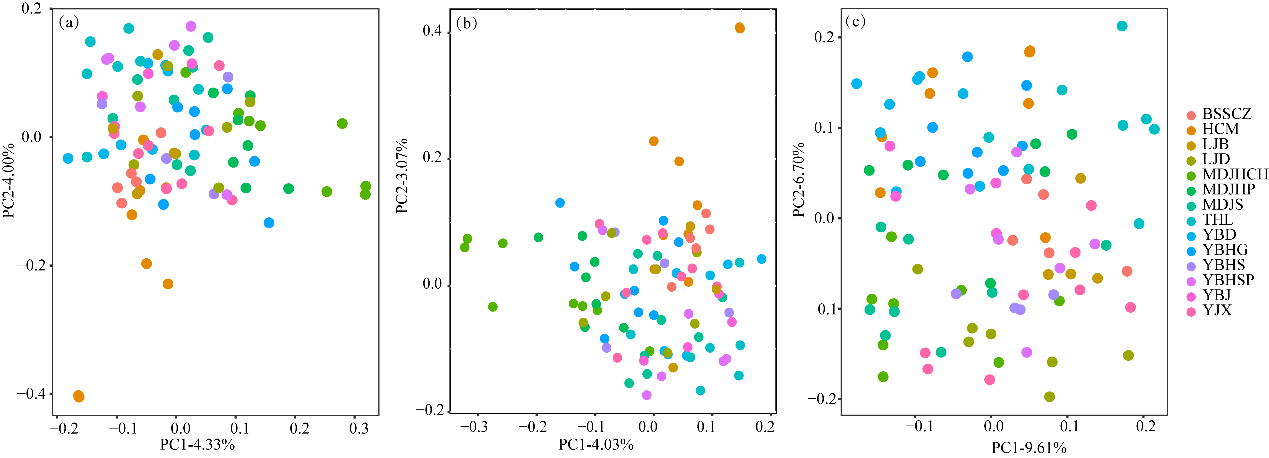
**

**Fig. S2.** Principal component analysis (PCA) of *T. cuspidata* by all (a), neutral (b) and outlier (c) SNPs.


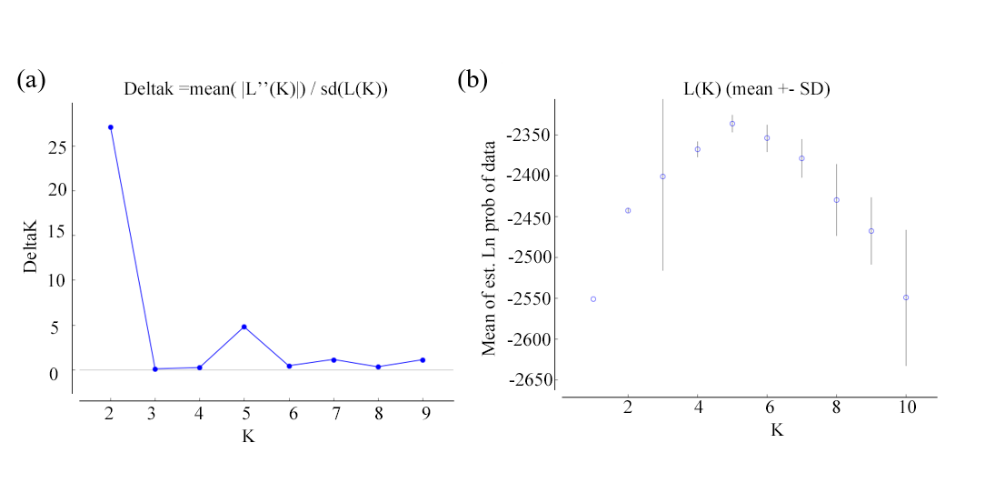


**Fig. S3.** Results of the STRUCTURE analysis in *T. cuspidata* by SSR dataset. (a) magnitude of △K as a function of K, with K-values ranging from one to ten, (b) likelihood of K for each value of K.

**
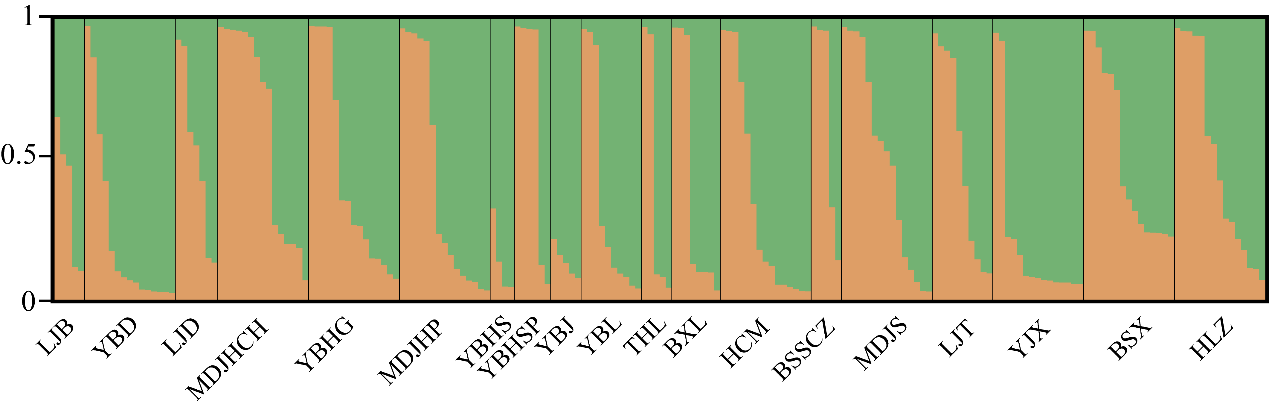
**

**Fig. S4.** Histogram of individuals assignments for K=2 by SSR dataset.


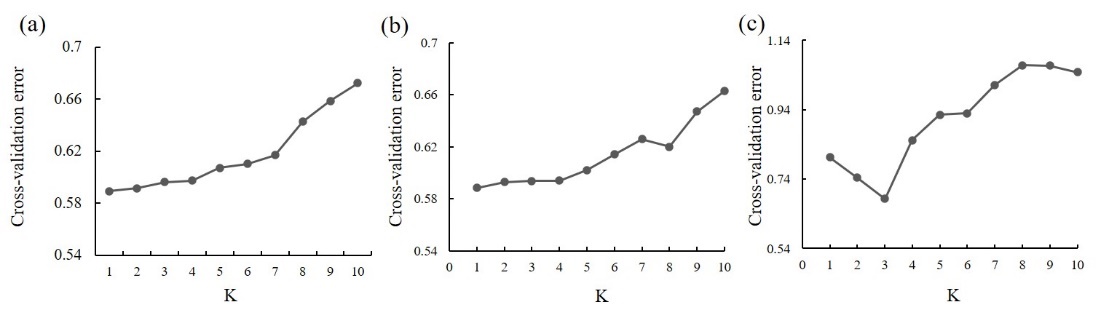


**Fig. S5.** Results of the ADMIXTURE analysis in *T. cuspidata* using RAD-seq dataset. Cross-validation (CV) of all (a), neutral (b) and outlier (c) SNPs derived using v = 5 folds.

**
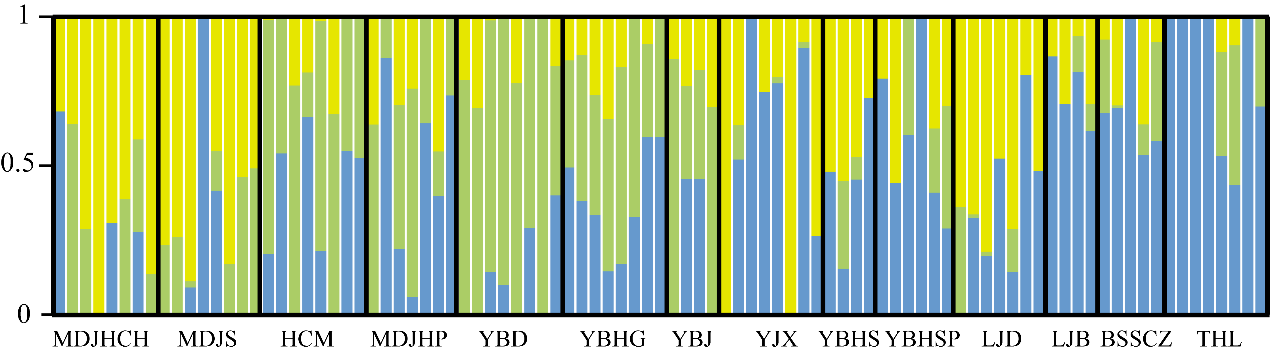
**

**Fig. S6.** Histogram of individuals assignments for K=3 by outlier SNPs.


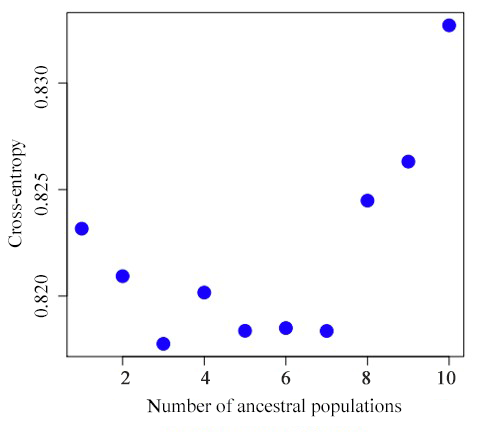


**Fig. S7.** Value of the cross-entropy criterion as a function of the number of populations in SNMF.

**
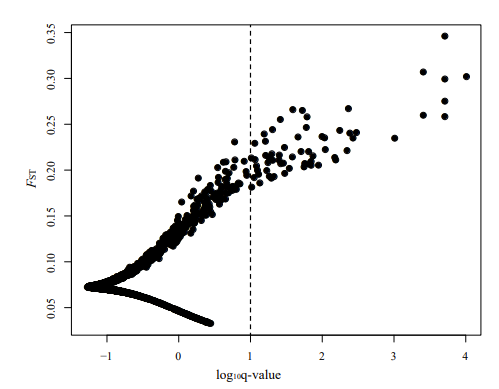
**

**Fig. S8.** Results for the outlier *F*_ST_ test detected SNPs for putative loci under selection by BayeScan, the dots to the right of the vertical dashed line represented the SNPs with outlier *F*_ST_ values that had experienced natural selection.

**
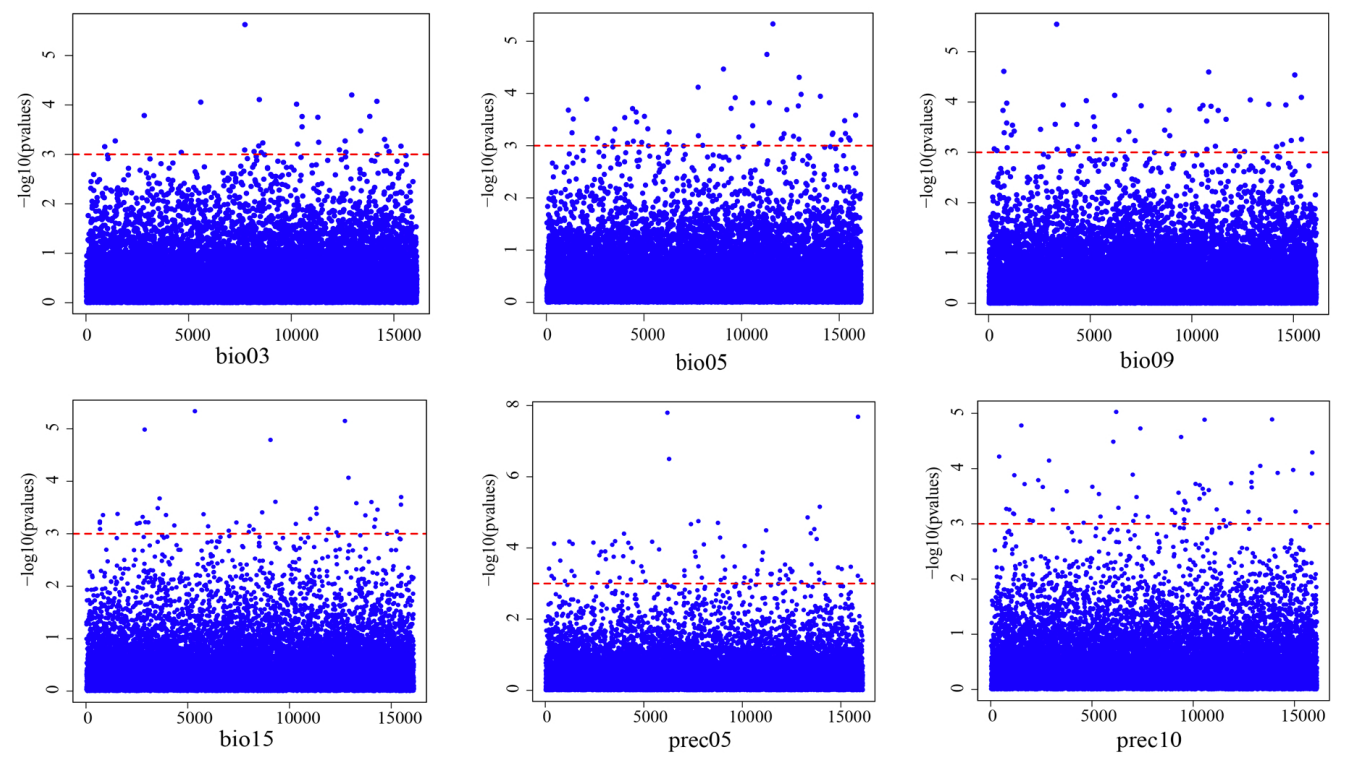
**

**Fig. S9.** Manhattan plot of SNPs called from the scale with six environment variables by LFMM. X-axis represents all SNPs and y-axis represents -log_10_ *p*-value for each SNP. Each blue dot in a Manhattan plot represents a SNP. Above the red line are SNPs significantly associated with climate variables.

**
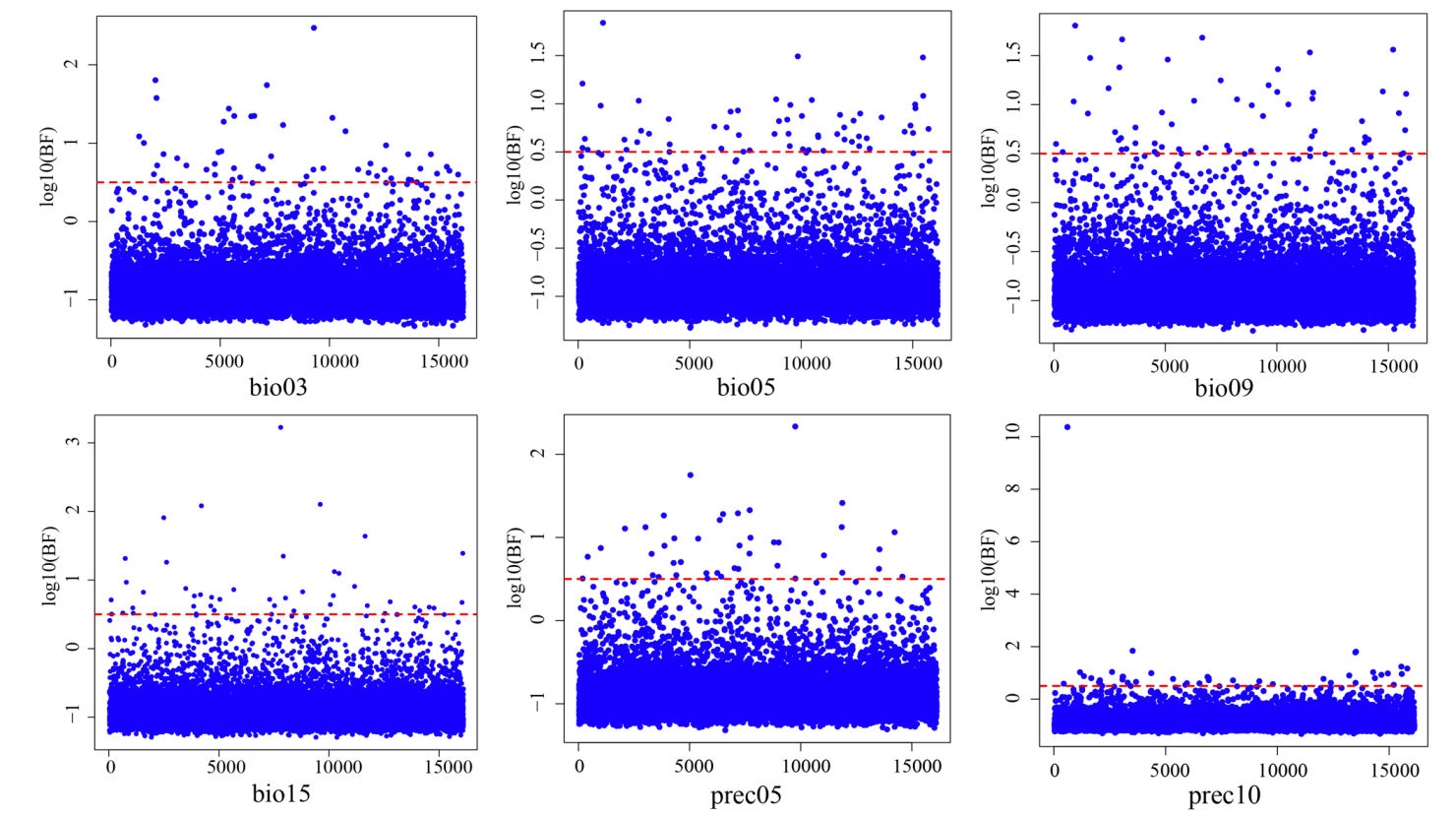
**

**Fig. S10.** Manhattan plot of SNPs called from the scale six environment variables by BayEnv. X-axis represents all SNPs and y-axis represents log_10_ (BF) for each SNP. Each blue dot in a Manhattan plot represents a SNP. Above the red line are SNPs significantly associated with climate variables.

**
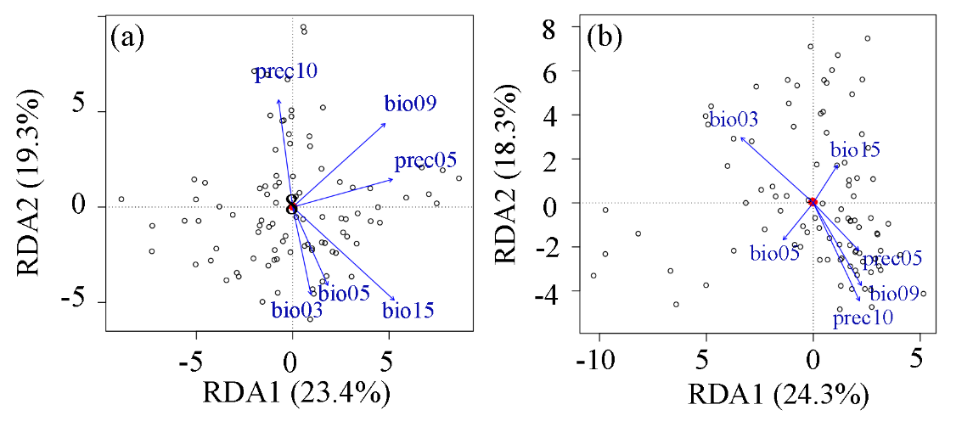
**

**Fig. S11.** Loadings of climate variables with first two axes based on all SNPs by RDA (a) and *p*RDA (b).

**
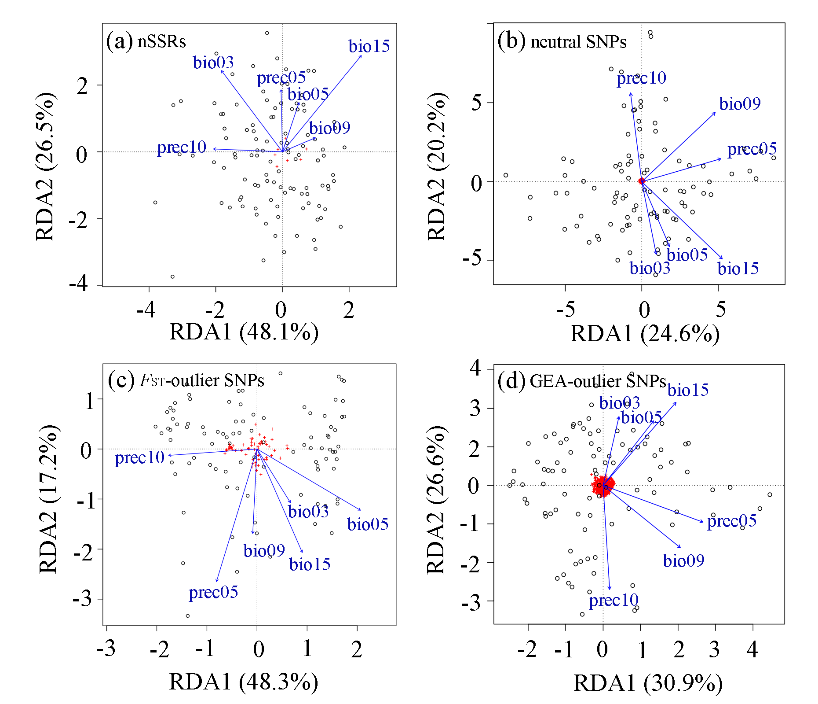
**

**Fig. S12.** Loadings of climate variables with first two axes based on neutral and non-neutral genetic variation by RDA.


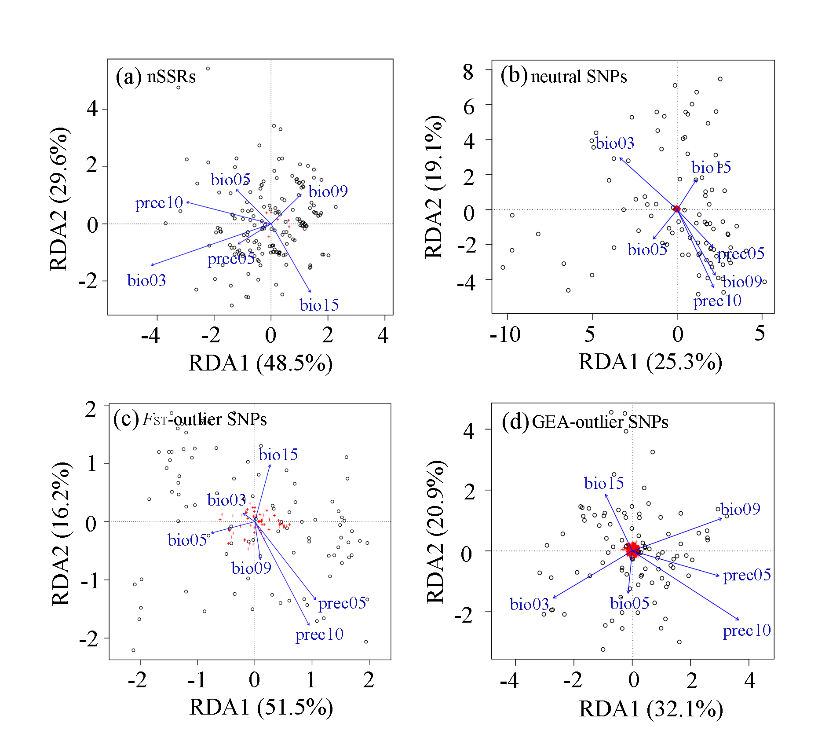


**Fig. S13.** Loadings of climate variables with first two axes based on neutral and non-neutral genetic variation by *p*RDA.

**Supplementary Tables**

**Table S2.** Seven pairs of nSSR primers were used for PCR amplifying in *T. cuspidata*.

| **Locus** | **Forward sequence** | **Reverse sequence** | **Fluorescent tags** | **Repeat** | **Length(bp)** | **Tm**  **(℃)** | **References** |
| --- | --- | --- | --- | --- | --- | --- | --- |
| Tax86 | CCCTAGGGTTGGTGGAATTT | TGTGGGAATCCATTTAAGCA | TAMRA | GT | 152-304 | 55 | Dubreuil et al.，2008 |
| T2 | AACGTTGTAAATCATTTGGACTCA | CGGCATGAAATAGGATCAAAC | FAM | AT | 138-184 | 53 | Cheng et al.，2015 |
| T20 | TCTTAGCCCTTTGGTTCTACACA | ATTCTAGAGGGTTGATGCGAGA | HEX | TC | 174-180 | 49 | Chenget al.，2015 |
| TC20343 | TGCAACCATGAATGTATTTGTACT | AGAGCATAAAGTCGGTTCGTT | ROX | AC | 166 | 60 | Kondo，2016 |
| TC23535 | CCTTACCCTTGTGGACGTGT | CCAAGCAGTGAAAAATTCAAGCA | TAMRA | AT | 108 | 60 | Kondo，2016 |
| TC35366 | CCAAAGGTGTGGGCTTAAGC | AACCATATCCCTCAGGTGCA | FAM | AT | 190-254 | 60 | Kondo，2016 |
| TC82541 | TGGAAAGGCATGAAGAGGGG | TCCTCTTGAGGTGCACCCTA | FAM | AG | 305-379 | 60 | Kondo，2016 |

**Table S4.** Genetic diversity of *T. cuspidata* based on all SNPs.

| **Population** | **N** | **P** | ***H*_o_** | ***H*_E_** | **π** |
| --- | --- | --- | --- | --- | --- |
| LJB | 4 | 0.79 | 0.32 | 0.28 | 0.32 |
| YBD | 8 | 0.79 | 0.28 | 0.29 | 0.31 |
| LJD | 7 | 0.79 | 0.26 | 0.29 | 0.31 |
| MDJHCH | 8 | 0.79 | 0.27 | 0.28 | 0.30 |
| YBHG | 8 | 0.78 | 0.31 | 0.30 | 0.32 |
| MDJHP | 7 | 0.80 | 0.24 | 0.26 | 0.29 |
| YBHS | 4 | 0.79 | 0.28 | 0.27 | 0.33 |
| YBHSP | 6 | 0.80 | 0.28 | 0.27 | 0.31 |
| YBJ | 4 | 0.81 | 0.31 | 0.26 | 0.30 |
| THL | 8 | 0.78 | 0.30 | 0.30 | 0.33 |
| HCM | 8 | 0.80 | 0.26 | 0.27 | 0.30 |
| BSSCZ | 5 | 0.81 | 0.27 | 0.26 | 0.29 |
| MDJS | 8 | 0.78 | 0.25 | 0.30 | 0.32 |
| YJX | 8 | 0.78 | 0.30 | .0.30 | 0.32 |
| YBL | 5 | - | - | - | - |
| BXL | 8 | - | - | - | - |
| LJT | 8 | - | - | - | - |
| BSX | 8 | - | - | - | - |
| HLZ | 8 | - | - | - | - |

N: number of individuals used for RAD-seq libraries; P, mean frequency of the most frequent allele at each locus; *H*_o_ and *H*_E_, Observed and expected heterozygosity; π, mean nucleotide diversity.

**Table S5.** Genetic diversity of *T. cuspidata* based on nSSRs dataset in 19 populations.

| **Population** | **N** | ***N*_A_** | ***N*_E_** | ***H*_o_** | ***H*_E_** |
| --- | --- | --- | --- | --- | --- |
| LJB | 5 | 2.43 | 1.71 | 0.43 | 0.34 |
| YBD | 15 | 3.57 | 2.13 | 0.42 | 0.46 |
| LJD | 7 | 2.71 | 2.23 | 0.31 | 0.47 |
| MDJHCH | 15 | 4.14 | 2.48 | 0.39 | 0.48 |
| YBHG | 15 | 3.00 | 1.90 | 0.29 | 0.34 |
| MDJHP | 15 | 3.57 | 2.17 | 0.38 | 0.43 |
| YBHS | 4 | 2.14 | 1.73 | 0.39 | 0.34 |
| YBHSP | 6 | 3.14 | 2.21 | 0.36 | 0.39 |
| YBJ | 5 | 2.29 | 1.71 | 0.46 | 0.34 |
| THL | 10 | 2.57 | 1.72 | 0.30 | 0.34 |
| HCM | 15 | 3.43 | 1.90 | 0.29 | 0.40 |
| BSSCZ | 5 | 2.29 | 1.94 | 0.26 | 0.28 |
| MDJS | 15 | 4.14 | 2.34 | 0.36 | 0.44 |
| YJX | 15 | 2.57 | 1.58 | 0.26 | 0.29 |
| YBL | 5 | 2.57 | 1.79 | 0.29 | 0.31 |
| BXL | 8 | 2.43 | 1.56 | 0.29 | 0.31 |
| LJT | 10 | 3.43 | 2.29 | 0.26 | 0.38 |
| BSX | 15 | 2.86 | 1.71 | 0.34 | 0.36 |
| HLZ | 15 | 3.43 | 1.92 | 0.33 | 0.38 |
| mean |  | 2.98 | 1.95 | 0.34 | 0.37 |

*N*_A_, No. of different alleles and *N*_E_, effective number of alleles; *H*_o_ and *H*_E_, Observed and expected heterozygosity

**Table S6.** Summary of number of outlier SNPs associated with environmental under putative selection identified by LFMM and BayEnv.

|  | **Bio03** | **Bio05** | **Bio09** | **Bio015** | **Prec05** | **Prec10** |
| --- | --- | --- | --- | --- | --- | --- |
| LFMM | 29 | 49 | 51 | 43 | 87 | 61 |
| BayEnv | 50 | 56 | 55 | 44 | 42 | 40 |
| Total | 79 | 105 | 105 | 87 | 129 | 101 |

**Table S7.** Summary and partitioning of the variance associated with climate and geographic variables by RDA in neutral and non-neutral genetic variation.

|  | **Neutral genetic variation** | | | | | | |  | **Non-neutral genetic variation** | | | | | | |
| --- | --- | --- | --- | --- | --- | --- | --- | --- | --- | --- | --- | --- | --- | --- | --- |
|  | **nSSRs** | | |  | **Neutral SNPs** | | |  | ***F*_ST_-outlier SNPs** | | |  | **GEA-outlier SNPs** | | |
|  | PVE | Eigenvalue | *P* |  | PVE | Eigenvalue | *P* |  | PVE | Eigenvalue | *P* |  | PVE | Eigenvalue | *P* |
| Climate | 5.3 | 1.8 | 0.002 |  | 10.63 | 1.71 | 0.001 |  | 23.93 | 4.51 | 0.001 |  | 17.95 | 3.13 | 0.001 |
| bio03 | 0.9 | 1.84 | 0.08 |  | 1.69 | 1.62 | 0.002 |  | 2.33 | 2.64 | 0.014 |  | 2.91 | 0.03 | 0.001 |
| bio05 | 0.54 | 1.1 | 0.36 |  | 1.55 | 1.49 | 0.001 |  | 5.52 | 6.24 | 0.001 |  | 2.94 | 3.08 | 0.001 |
| bio09 | 0.62 | 1.26 | 0.278 |  | 2.18 | 2.09 | 0.001 |  | 3.3 | 3.73 | 0.001 |  | 4.49 | 4.7 | 0.001 |
| bio15 | 1.77 | 3.61 | 0.001 |  | 1.86 | 1.79 | 0.001 |  | 2.99 | 3.38 | 0.002 |  | 3.03 | 3.18 | 0.001 |
| prec05 | 0.44 | 0.89 | 0.526 |  | 1.63 | 1.56 | 0.001 |  | 7.33 | 8.29 | 0.001 |  | 2.91 | 3.05 | 0.001 |
| prec10 | 1.03 | 2.1 | 0.055 |  | 1.72 | 1.66 | 0.004 |  | 2.46 | 2.79 | 0.228 |  | 1.67 | 1.76 | 0.004 |

Abbreviations: PVE, percentage of explained variance

**Table S8.** Summary and partitioning of the variance associated with climate and geographic variables based on redundancy analysis (RDA) and partial RDA (*p*RDA) for all SNPs.

|  | | **PVE** | **Eigenvalue** | ***P*** |
| --- | --- | --- | --- | --- |
| Geography | 3.43 | | 1.68 | 0.001 |
| Climate | 11.01/10.16 | | 1.77/1.67 | 0.001/0.001 |
| bio03 | | 1.75/1.85 | 1.7/1.81 | 0.001/0.001 |
| bio05 | | 1.62/1.38 | 1.56/1.36 | 0.001/0.004 |
| bio09 | | 2.28/1.64 | 2.21/1.61 | 0.001/0.001 |
| bio15 | | 1.94/1.63 | 1.87/1.6 | 0.001/0.001 |
| prec05 | | 1.7/1.94 | 1.64/1.91 | 0.001/0.001 |
| prec10 | | 1.72/1.72 | 1.66/1.69 | 0.001/0.001 |

The values on the left and right sides of the slash represent the RDA and *p*RDA results, respectively.

**Table S9.** Summary of the genetic offset values for all SNPs and outlier SNPs in 2070.

| **Genetic offset value** |  | | **RCP 2.6** | |  |  | | **RCP 8.5** | |
| --- | --- | --- | --- | --- | --- | --- | --- | --- | --- |
|  | **All SNPs** | ***F*_ST_-outlier SNPs** | | **GEA-outlier SNPs** |  | **All SNPs** | ***F*_ST_-outlier SNPs** | | **GEA-outlier SNPs** |
| Minimum | 0.20 | 0.56 | | 0.27 |  | 0.20 | 0.56 | | 0.27 |
| Maximum | 1.46 | 4.06 | | 1.72 |  | 1.56 | 4.09 | | 1.84 |
| Mean | 1.17 | 3.28 | | 1.31 |  | 1.19 | 3.47 | | 1.33 |

**Table S10.** Summary of RONA was calculated for RCP 2.6 and RCP 8.5 in *T. cuspidata* by future climatic predictions for 2070.

|  | **RCP 2.6** | | |  | **RCP 8.5** | | |
| --- | --- | --- | --- | --- | --- | --- | --- |
|  | **Prec05** | **Bio03** | **Prec10** |  | **Prec05** | **Bio03** | **Prec10** |
| MDJHCH | 0.0310 | 0.0218 | 0.0530 |  | 0.1591 | 0.1074 | 0.2238 |
| MDJS | 0.1229 | 0.0774 | 0.1219 |  | 0.2392 | 0.1402 | 0.5209 |
| HCM | 0.2247 | 0.0789 | 0.0000 |  | 0.3433 | 0.1710 | 0.3968 |
| MDJHP | 0.1799 | 0.0435 | 0.2045 |  | 0.2022 | 0.0991 | 0.2398 |
| YBD | 0.2029 | 0.0635 | 0.2358 |  | 0.3166 | 0.2186 | 0.0000 |
| YBHG | 0.2124 | 0.0614 | 0.1018 |  | 0.2150 | 0.0988 | 0.0278 |
| YBJ | 0.1686 | 0.1072 | 0.2059 |  | 0.3426 | 0.1496 | 0.4097 |
| YJX | 0.2359 | 0.0000 | 0.2806 |  | 0.1977 | 0.0737 | 0.3536 |
| YBHS | 0.1740 | 0.0706 | 0.2703 |  | 0.1357 | 0.1545 | 0.3400 |
| YBHSP | 0.2566 | 0.0624 | 0.2636 |  | 0.1580 | 0.2017 | 0.3457 |
| LJD | 0.1464 | 0.0000 | 0.2015 |  | 0.3323 | 0.0000 | 0.4614 |
| LJB | 0.0000 | 0.1004 | 0.2078 |  | 0.2068 | 0.1191 | 0.0526 |
| BSSCZ | 0.1988 | 0.0850 | 0.2145 |  | 0.2795 | 0.1260 | 0.2872 |
| THL | 0.2045 | 0.0425 | 0.3696 |  | 0.0013 | 0.0525 | 0.0277 |
| SNPs | 349 | 309 | 300 |  | 349 | 309 | 300 |
| MinR^2^ | 0.0000 | 0.0000 | 0.0000 |  | 0.0000 | 0.0000 | 0.0000 |
| MaxR^2^ | 0.5806 | 0.4924 | 0.4804 |  | 0.5900 | 0.7005 | 0.4461 |
| AverageR^2^ | 0.0673 | 0.0605 | 0.0693 |  | 0.0931 | 0.1042 | 0.0573 |
